# Identifying Crucial Parameter Correlations Maintaining Bursting Activity

**DOI:** 10.1371/journal.pcbi.1003678

**Published:** 2014-06-19

**Authors:** Anca Doloc-Mihu, Ronald L. Calabrese

**Affiliations:** Department of Biology, Emory University, Atlanta, Georgia, United States of America; Université Paris Descartes, Centre National de la Recherche Scientifique, France

## Abstract

Recent experimental and computational studies suggest that linearly correlated sets of parameters (intrinsic and synaptic properties of neurons) allow central pattern-generating networks to produce and maintain their rhythmic activity regardless of changing internal and external conditions. To determine the role of correlated conductances in the robust maintenance of functional bursting activity, we used our existing database of half-center oscillator (HCO) model instances of the leech heartbeat CPG. From the database, we identified functional activity groups of burster (isolated neuron) and half-center oscillator model instances and realistic subgroups of each that showed burst characteristics (principally period and spike frequency) similar to the animal. To find linear correlations among the conductance parameters maintaining functional leech bursting activity, we applied Principal Component Analysis (PCA) to each of these four groups. PCA identified a set of three maximal conductances (leak current, 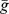
_Leak_; a persistent K current, 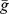
_K2_; and of a persistent Na+ current, 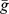
_P_) that correlate linearly for the two groups of burster instances but not for the HCO groups. Visualizations of HCO instances in a reduced space suggested that there might be non-linear relationships between these parameters for these instances. Experimental studies have shown that period is a key attribute influenced by modulatory inputs and temperature variations in heart interneurons. Thus, we explored the sensitivity of period to changes in maximal conductances of 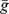
_Leak_, 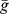
_K2_, and 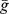
_P_, and we found that for our realistic bursters the effect of these parameters on period could not be assessed because when varied individually bursting activity was not maintained.

## Author Summary

Central pattern-generating networks (CPGs) must be remarkably robust, maintaining functional rhythmic activity despite fluctuations in internal and external conditions. Recent experimental evidence suggests that this robustness is achieved by the coordinated regulation of many membrane and synaptic current parameters. Experimental and computational studies showed that linearly correlated sets of such parameters allow CPG neurons to produce and maintain their rhythmic activity. However, the mechanisms that allow multiple parameters to interact, thereby producing and maintaining rhythmic single cell and network activity, are not clear. Here, we use a half-center oscillator (HCO) model that replicates the electrical activity (rhythmic alternating bursting of mutually inhibitory interneurons) of the leech heartbeat CPG to investigate potential relationships between parameters that maintain functional bursting activity in the HCOs and the isolated component neurons (bursters). We found a linearly correlated set of three maximal conductances that maintains functional bursting activity similar to the animal in burster model instances, therefore increasing robustness of bursting activity. In addition, we found that bursting activity was very sensitive to individual variation of these parameters; only correlated changes could maintain the activity type.

## Introduction

Vital adaptive rhythmic behaviors such as breathing and heartbeat in invertebrates are produced by central pattern-generating networks (CPGs). Beside their inherent importance in pacing rhythmic movements, CPGs represent fertile test beds for understanding neuronal network dynamics because of the robustness of their activity patterns even in reduced experimental preparations [1], [2], [3]. The combination of the intrinsic electrical properties of the component neurons and their synaptic interactions within a CPG produces their rhythmic activity [1]. To maintain functional rhythmic activity, the CPG neurons and networks must be remarkably robust regardless of changing internal and external conditions. Recent experimental evidence suggests that animals show robust responses to modulation and environmental perturbations (e.g., large temperature changes [4], [5], [6]). Modeling studies have begun to address the mechanisms underlying the robustness in activity type. For example, Goldman et al. [7] tested a model neuron over a wide range of parameters and found that activity type was robust to certain changes in parameters but very sensitive to other changes.

Bursting activity in CPGs [1], [8], [9], [10] is characterized by intervals of repetitive spiking separated by intervals of quiescence. Autonomously bursting neurons are common components of CPGs [3]. Half-center oscillators (HCOs), which consist of reciprocally inhibitory neurons (often autonomous bursters), are one of the most prevalent circuit building blocks of CPGs that are thought to assure robust alternating bursting [3], [10]. Studies of HCOs show that they can display a wide range of bursting activity when the parameters controlling intrinsic membrane properties and synaptic interactions of the neurons are varied [1], [8], . The analysis of this basic circuit building block has helped researchers understand how bursting activity is generated and how motor patterns are controlled by the nervous system. One CPG that is particularly well understood controls heartbeat in leeches [2]. The heartbeat period is regulated by a variety of environmental (e.g. changes in temperature) and physiologic (brought on by locomotor movements like swimming) inputs. When temperature increases [14], the burst period of the heartbeat CPG decreases [15]. Similarly, when the animals swim, the swim CPG is active and the heartbeat CPG burst period decreases [14]. Therefore period is an important regulated characteristic of this CPG.

Recent experimental and modeling analyses of bursting activity indicate that the parameters (specifically the maximal conductances of specific ion channels) influencing bursting activity show 3–5 fold variation from animal to animal or model instance to model instance but that there are relationships (linear or non-linear) between parameters [16]. For example, electrophysiological and molecular studies in stomatogastric neurons [17], [18], [19] found pairwise and four-way linear correlations between the parameters. These studies suggest that the functional activity of a given neuron may reside in the set of parameter correlation rules it maintains rather than in the value of any particular parameter. In addition, such correlations were also found in model solution spaces obtained by parameter space exploration of biologically realistic models [20], [21], [22]. Many studies, both experimental [23], [24] and computational [11], have provided evidence that linearly correlated sets of parameters (intrinsic and synaptic properties of neurons) allow CPG neurons to produce and maintain their rhythmic activity. To establish parameters relationships, some studies have used new visualizations (e.g., NDVis, parameterscape) [25], [26], [9], while others have used mathematical methods (e.g., regression, discriminant analysis) [27], [28]. However, it is still unclear, how multiple parameters interact to produce and maintain the rhythmic single cell and network activity. Our study here focuses on how intrinsic membrane and synaptic parameters interact to maintain functional bursting activity in HCO and in burster model neurons from the leech heartbeat CPG.

For our study, we used the HCO computational model of Hill et al. [29], which was successfully developed to replicate the electrical activity (rhythmic alternating bursting of mutually inhibitory neurons) of the oscillator (HN) interneurons of the leech heartbeat CPG under a variety of experimental conditions. This HCO model consists of a two reciprocally inhibitory model HN interneurons, represented as single isopotential electrical compartments with Hodgkin and Huxley [30] type intrinsic and synaptic membrane conductances. Each compartment contains 8 voltage-dependent currents, five inward currents I_Na_ - a fast Na^+^ current, I_P_ - a persistent Na^+^ current, I_CaF_ - a rapidly inactivating low-threshold Ca current, I_CaS_ - a slowly inactivating low-threshold Ca current, I_h_ - a hyperpolarization-activated cation current) and three outward currents (I_K1_ - a delayed rectifier-like K current, I_K2_ - a persistent K current, I_KA_ - a fast transient K current). The model has two types of inhibitory synaptic transmission between the two interneurons: graded transmission (I_SynG_) and spike-mediated transmission (I_SynS_). The maximal conductances (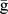) of each of the membrane and synaptic currents and the leak reversal potential (E_Leak_) are free parameters in the model.

A comprehensive analysis of parameter relationships in the complete, canonical HCO neuron model presents a computational and theoretic challenge. To systematically explore the parameter space of the HCO and corresponding burster models, in our previous work [31], we simulated about 10.5 million model instances, whose characteristics we recorded into a database named HCO-db [31], [32]. The simulations were obtained by co-varying a carefully selected set of parameters that single parameter variation analyses showed were crucial in establishing bursting and controlling burst period [29]. These parameters comprise the maximal conductances 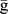
_SynS_, 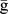
_SynG_, 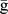
_P_, 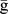
_K2_, 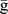
_h_, 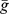
_CaS_ and 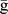
_Leak_ (varied across of 0%, 25%, 50%, 75%, 100%, 125%, 150%, and 175% of their canonical values), and E_Leak_ (varied across −70, −65, −60, −55, and −50 mV values) in all possible combinations. All simulated instances were classified into separate groups showing the same electrical activity. Our HCO-db is a very efficient tool for querying the simulated HCO model instances for finding potential parameter relationships.

In this study, we focused only on the four groups of instances from our HCO-db database showing functional leech bursting characteristics, HCOs, realistic HCOs, bursters, and realistic bursters. A HCO instance has two model interneurons each showing bursting activity with at least two bursts in a 40 s time interval, and has the following characteristics: each of its bursts has normal spikes (See Definitions), a small variation of period, a relative phase in the range of (0.45–0.55), and at least one synaptic component present (either 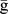
_SynS_≠0, or 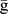
_SynG_≠0, or both 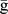
_SynS_≠0 and 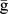
_SynG_≠0). A realistic HCO instance is a HCO that showed realistic bursting corresponding to that observed in leech oscillator heart interneurons (period between 5–15 s, average spike frequency between 8–25 Hz, and duty cycle between 50–70%). An isolated neuron instance (isolated neuron) has two identical interneurons (though started with different initial conditions, but otherwise identical), and no synaptic interaction (i.e., 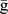
_SynS_ = 0 and 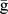
_SynG_ = 0). A burster instance is an isolated neuron instance for which both neurons had at least two bursts, each with normal spikes, and regular periods (as defined above for the HCOs). Note that burster instances can be thought of as being HCOs with no synaptic connections. A realistic burster instance is a burster instance that showed realistic bursting corresponding to isolated leech oscillator heart interneurons (period between 5–15 s, and average spike frequency between 8–25 Hz). Notice that realistic instances are a subgroup of either HCOs or bursters, and in our discussion here, unless specifically indicated, the HCO and burster groups include their subgroup of realistic instances.

We applied Principal Component Analysis (PCA) to automatically find the potential existing linear correlations between the parameters maintaining functional activity. The results returned by PCA identified three maximal conductances (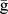
_P_, 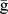
_K2_, and 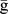
_Leak_) that correlate linearly for the bursters and the realistic bursters, and showed that for the HCOs and realistic HCOs there were no linear correlations between the parameters, but visualizations in a reduced space suggested that non-linear relationships between parameters might exists for these instances. In addition, we found that the bursting activity of the burster instances was very sensitive to variations in 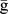
_P_, and 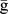
_Leak_ and to a lesser extent 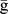
_K2_.

## Results

### Sustaining realistic bursting in HCOs and bursters

In previous work [31], our classification algorithm identified 1,202,139 (11.6%) HCO model instances as HCOs and 424 isolated neuron model instances as bursters (0.26% of the isolated neurons). To generate the *realistic* instance populations, we queried our database, HCO-db [32] with the criteria given in our definitions (see Methods) for the realistic HCO instances and realistic burster instances. We recorded the results of these queries into two separate views (MySQL Views) to facilitate and speed queries involving these groups in the future. We obtained 99,066 instances (8.2% of the HCOs) in the group of realistic HCOs, and 307 instances (72.4% of the bursters) in the group of realistic burster instances.

Out of 424 bursters, 263 produce realistic HCOs (1,055 instances) and 419 produce HCOs (21,303 instances) when coupled with inhibitory synapses. The number of HCO instances exceeds the number of burster instances because multiple values of synaptic conductance (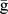
_SynS_ or 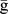
_SynG_) give rise to HCO instances for each burster instance. Of the 424 bursters, 307 are realistic bursters, and 238 of these produce 990 realistic HCOs (instances) when coupled with inhibitory synapses. That is, 25 bursters (out of 117 bursters that are not realistic) produced 65 realistic HCOs. All 307 realistic bursters produce HCOs when coupled with inhibitory synapses (16,805 HCO instances).

The vast majority of HCOs in the database are not composed of bursters isolated neurons but of spiking isolated neurons. For example, among 99,066 total realistic HCOs, only 1,055 (1.06%) were composed of bursters (263 instances) (including 990 realistic HCOs that were composed of realistic bursters (238 instances)) as stated above, but 94,487 (95.37%) were composed of spiking isolated neurons (12,443 instances) and 3,524 (3.56%) were composed of neurons classified as either bistable isolated neurons (3,096 HCOs from 820 isolated neuron instances), as irregular isolated neurons (368 HCOs from isolated neuron 55 instances), as silent isolated neurons (58 HCOs from 28 isolated neuron instances) or as plateau neurons (2 HCOs from 2 isolated neuron instances).Thus realistic HCOs could also consist of irregular (irregular bursters or irregular tonic firers), silent, or even bistable neurons. Previous work from our group shows that our burster instances have a high propensity for multistability and that mutual inhibition makes multistability much less prevalent [33]. Although we have not tested this idea systematically, we suspect that such multistability is present in the other classes of isolated neuron instances.

**Figure 1:**
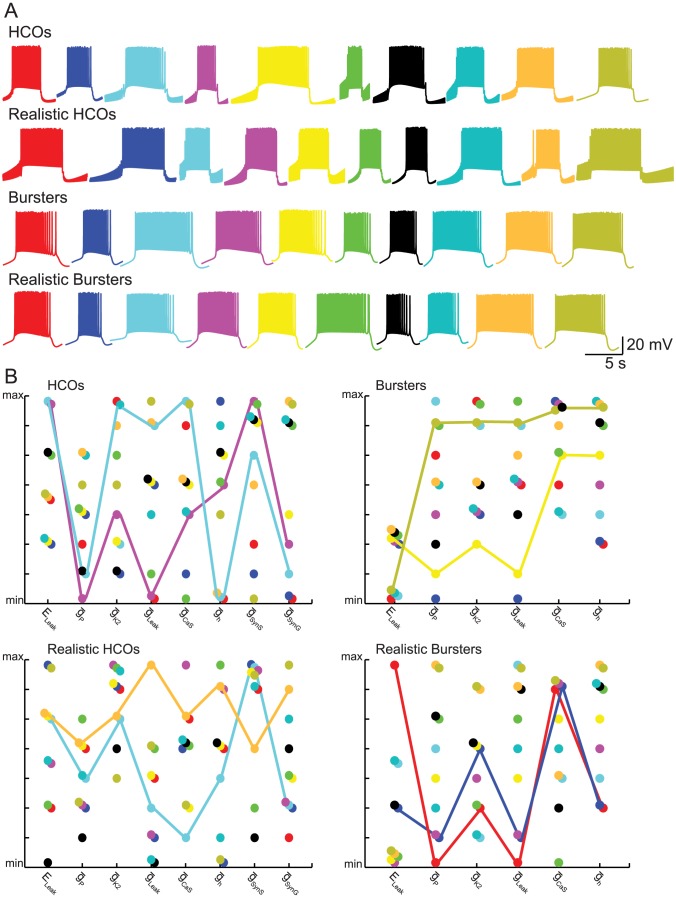
Activity and model parameters of ten instances, chosen at random from within each of the four groups defined in the text. (**A**) One cycle period of the voltage trace of each of ten random instances from each group. (**B**) Parameter values of the ten instances in each group shown in panel **A**, with corresponding colors. A colored line connects the parameters of the same instance. The parameter values were normalized for uniform scaling. Lines show very different patterns of parameter values of two instances despite having similar activity. Notice that the HCO and the burster instances shown might include the instances that were classified as realistic (HCOs and bursters, respectively).

Figure 1 shows the activity of ten randomly selected instances from each of the four groups of interest, HCOs, realistic HCOs, bursters, and realistic bursters. The figure shows that the instances within each group display different combinations of parameter values despite having similar bursting activity. For example, the two instances shown in turquoise and orange from the realistic HCO group have the same period (Figure 1A) and yet their parameters combinations are very different (colored connected lines Figure 1B).

In addition, Figure 1 illustrates that the parameter values of the instances within each group have wide ranges for almost every parameter. Some patterns seem to emerge in the parameter ranges that support our four categories of bursting. For example, both HCO and realistic HCO instances are possible without h-current (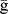
_h_ = 0), while both bursters and realistic bursters require at least 50% of the canonical level of 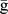
_h_.

**Figure 2:**
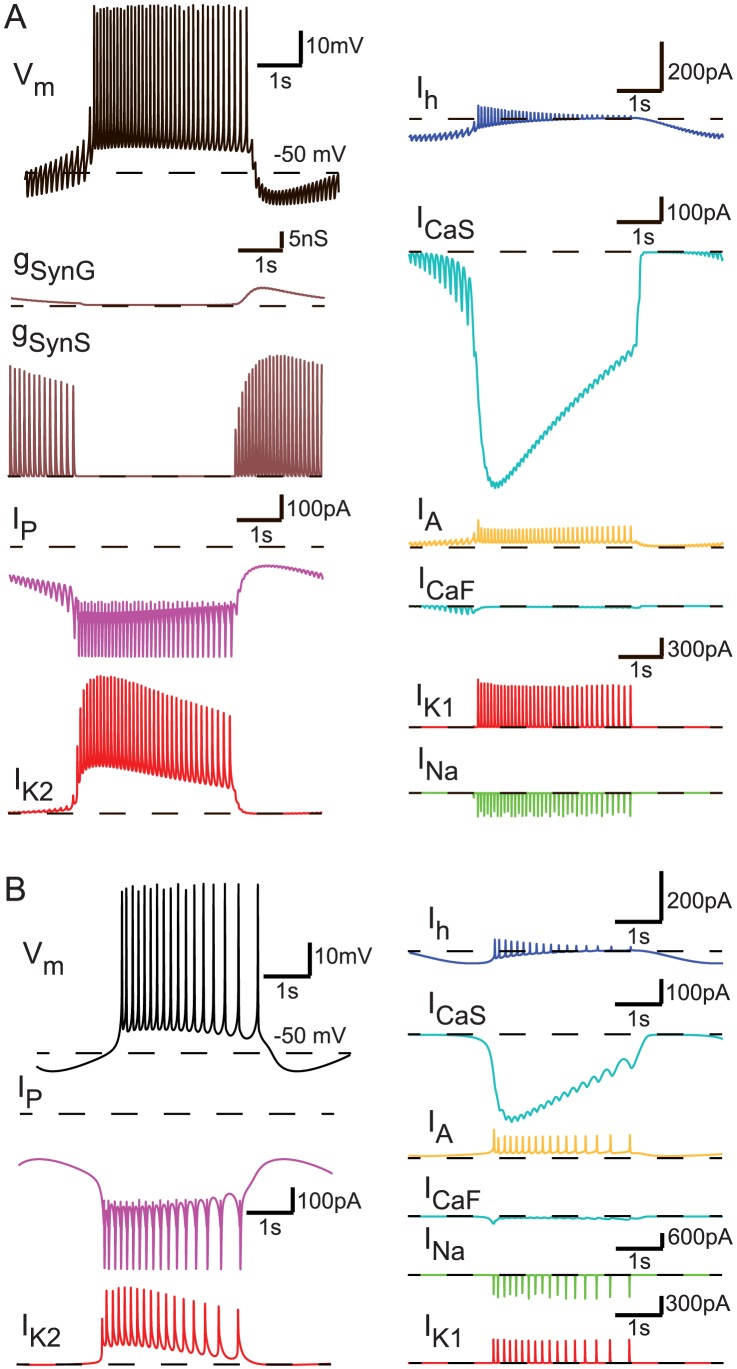
Intrinsic currents and synaptic conductances of two realistic instances presented in Figure 1. The traces shown are color coded for easy comparison across the two instances. (**A**) Data corresponding to the realistic HCO instance colored in orange in Figure 1 (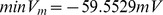). (**B**) Data corresponding to the realistic burster colored in black in Figure 1 (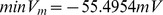).

Figure 2 shows the intrinsic currents and synaptic conductances of two realistic instances randomly chosen from the ones presented in Figure 1 (the realistic HCO shown in orange and the realistic burster shown in black). Both instances replicate (with respect to period and spike frequency, and for the realistic HCOs duty cycle) the oscillatory activity of leech HN interneurons, when coupled (Figure 2A) and in isolation (Figure 2B). For the realistic HCO instance the leak reversal potential is E_Leak_ = −55 mV (with *minV_m_* = −59.5529*mV*), and for the realistic burster is E_Leak_ = −65 mV (with *minV_m_* = −55.4954*mV*). During the inhibited phase (interburst interval) of the burst cycle in the HCO, the hyperpolarization-activated cation current, I_h_, slowly activates, depolarizing the inhibited neuron toward a burst (escape). The persistent Na^+^ current, I_P_, also helps in depolarizing the inhibited neuron. The burst is formed by the rapid activation of slowly inactivating low threshold Ca^2+^ current, I_CaS_ (I_CaF_ is very small in most instances) and the inactivation of I_CaS_ leads to its gradual decline leading to a reduced spike frequency and less inhibition of the opposite neuron (release). During the burst I_P_ sustains depolarization and a baseline spike frequency, and the outward currents I_K2_ and I_Leak_ oppose; during the inhibited phase I_P_ is opposed by the I_Leak_ and the synaptic currents. The balance between I_P_, I_Leak_, and I_K2_ appears crucial for maintaining the excitability of the system and setting the membrane potential about which the system oscillates (n.b. the −50 mV line). The spike currents I_Na_ and I_K2_ (I_KA_ is small in most instances) do not directly participate in burst formation but simply provide a baseline of excitability against which the more persistent currents act. When the neurons are isolated (Figure 2B), done in the HCO model by setting the maximal conductances of both synapses to 0 (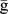
_SynS_ = 0 and 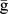
_SynG_ = 0), basically the same interactions apply except that only I_Leak_ can oppose I_P_ during the interburst interval and hyperpolarize the membrane potential sufficiently to activate I_h_ (n.b. −50 mV line). The lack of inhibition leads to the apparent requirement for a relatively hyperpolarized E_Leak_ in burster instances [3]. Figures 1 and 2 illustrate the complex interactions of the membrane and synaptic currents and they also suggest potential interactions. For example, for both instances shown in Figure 2, the maximal conductances, 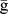
_P_ and 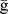
_K2_ (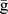
_P_ = 100%, 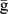
_K2_ = 125% for the realistic HCO, and 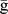
_P_ = 125%, 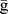
_K2_ = 100% for the realistic burster), have values close to each other, and 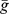
_Leak_ is large (175% and 150%, respectively). Is this anecdotal correlation a potential mechanism in the HCO model to maintain realistic (similar to animal) bursting activity? Next, we considered the influence of two parameters at the same time on the activity type of our four groups of interest.

### Visualizing interactions in pairwise parameter variations

**Figure 3:**
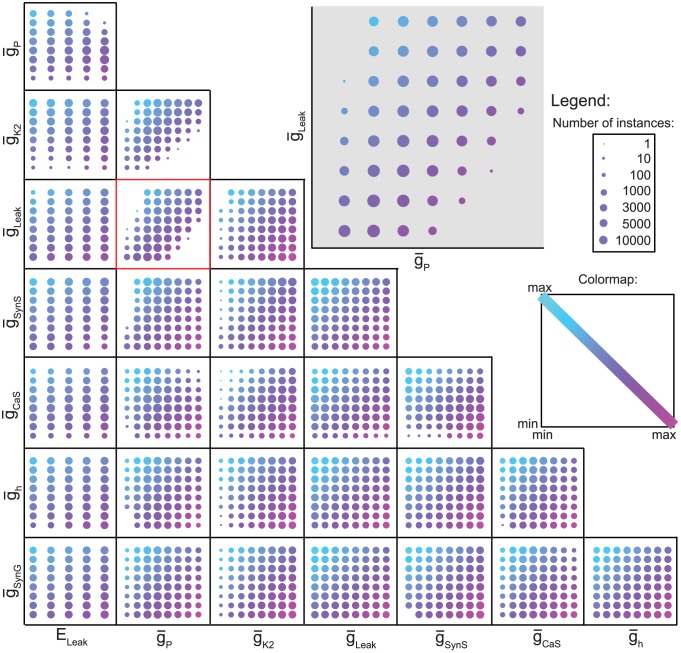
Pair-wise parameter variations for the realistic HCO instances. Plot of all instances within the realistic HCO group projected onto the 2D space given by two parameters. The first parameter is shown on x axis. The second parameter is shown on y axis. Both parameters are color coded, using shades of magenta on x axis and of turquoise on y axis, from dark shades for low values (−70 mV for E_Leak_ and 0% for the other parameters) to light shades for high values (−50 mV for E_Leak_.and 175% for the other parameters). The number of instances projected onto each point in the space is shown by the size of the circle surrounding it. To be able to visualize all the points (as there are many points with very few instances and many points with thousands of instances), we used the natural logarithm to adjust the size of the points (formula used: 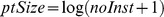, we added a value of 1 to be able to show a point with one instance on the plot). The legend shows the size of each point on the plot and its corresponding real number of instances. The highlighted subplot of 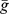
_Leak_ vs. 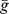
_P_ shows that there are exclusive zones of high 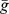
_Leak_ and low 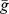
_P_ and of low 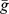
_Leak_ and high 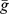
_P_, which do not support realistic HCOs.

**Figure 4:**
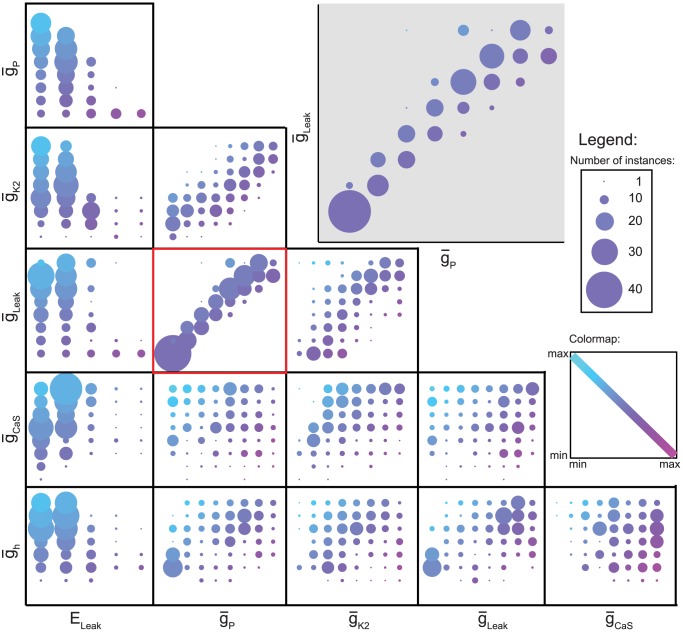
Pair-wise parameter variations for the realistic burster instances. Plot of all instances within the realistic burster group projected onto the 2D space given by two parameters. The first parameter is shown on x axis. The second parameter is shown on y axis. Both parameters are color coded, using shades of magenta on x axis and of turquoise on y axis, from dark shades for low values (−70 mV for E_Leak_ and 0% for the other parameters) to light shades for high values (−50 mV for E_Leak_.and 175% for the other parameters). The number of instances projected onto each point in the space is shown by the size of the circle surrounding it. The highlighted subplot of 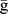
_Leak_ vs. 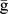
_P_ shows a positive correlation between 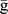
_Leak_ and 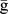
_P_ required to produce realistic bursters.

To explore visually the relationships existing between two parameters and a group of instances, we plotted the number of instances within a group versus all the possible values for two parameters. Several different methods were tried to make these plots, e.g., Supplemental Material Figure S2A and S2B, but we settled on the methods of Figures 3 and 4. These plot the number of instances as the size of each point and the two parameters on the x and y axes and cover all parameter pairs for our realistic groups of HCOs and bursters (similar but more populated plots for HCOs and bursters were obtained - data not shown).

Figure 3 shows these two-parameter plots for the realistic HCOs and illustrates the problems with a pair-wise approach because very little structure is apparent in any of the plots, but there were some exceptions. The plot of 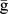
_Leak_ vs. 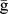
_P_ shows that there are exclusive zones of high 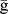
_Leak_ and low 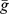
_P_ and of low 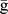
_Leak_ and high 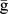
_P_, which do not support realistic HCOs. Similarly the plot of 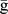
_K2_ vs. 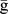
_P_ shows that there is a small exclusive zone of high 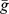
_K2_ and zero 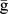
_P_ and a large exclusive zone of low 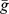
_K2_ and high 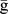
_P_, which do not support realistic HCOs. In general low 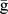
_P_ does not support realistic HCOs and middle values of 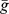
_P_ do support realistic HCOs and the absence of 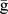
_P_ did not appear to limit the number of realistic HCO instances.

Figure 4 shows these two-parameters plots for the realistic bursters and reveal considerably more structure. First a non-zero 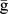
_h_ was required to produce realistic bursters and the next smallest values supported very few instances. The largest number of instances (28) was obtained for 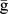
_h_ = 150% and more negative values of E_Leak_ (−70, −65 mV). There appears to be a positive correlation between 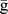
_Leak_ and 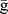
_P_ required to produce realistic bursters and similarly, but in a looser way, between 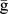
_K2_ and 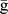
_P,_ and between 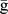
_Leak_ and 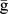
_K2_. Most notably, more positive values of E_Leak_, and low values of 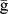
_CaS_ greatly restrict the number of realistic burster instances.

These pairwise parameter variation plots suggest potential parameter relationships between more than two parameters for us to investigate in our database using more rigorous mathematical methods to identify all potential linear relationships influencing activity type.

### Exploring linear relationships between parameters with PCA

**Figure 5:**
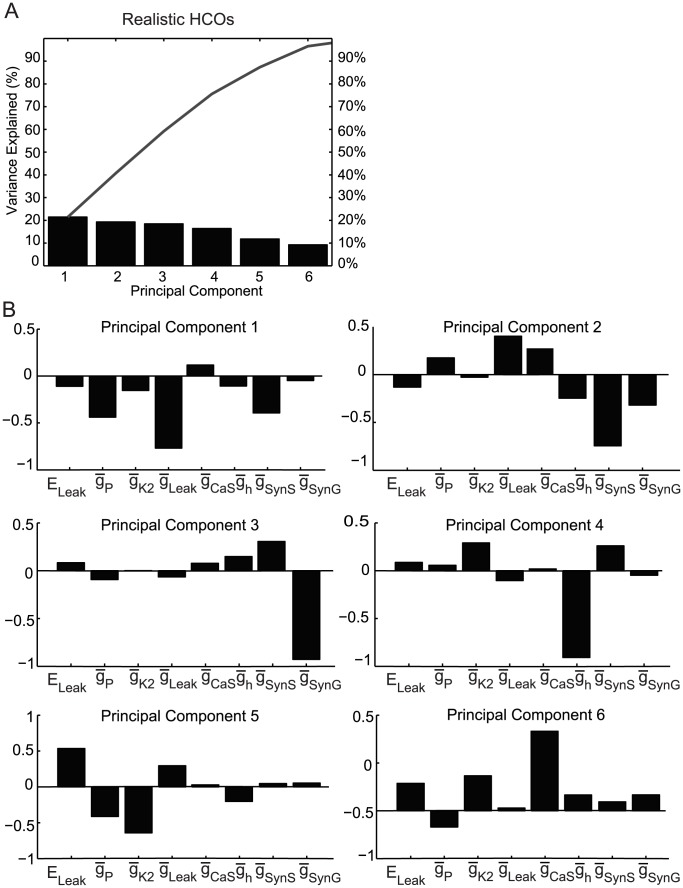
Principal components for the realistic HCOs. (**A**) The most important principal components for the group are the first six components with their sum of variance >95%. The line above the bars shows the cumulative percentage of the variance. (**B**) The coefficients of the linear combinations of the parameters that generate these first six (principal) components.

**Figure 6:**
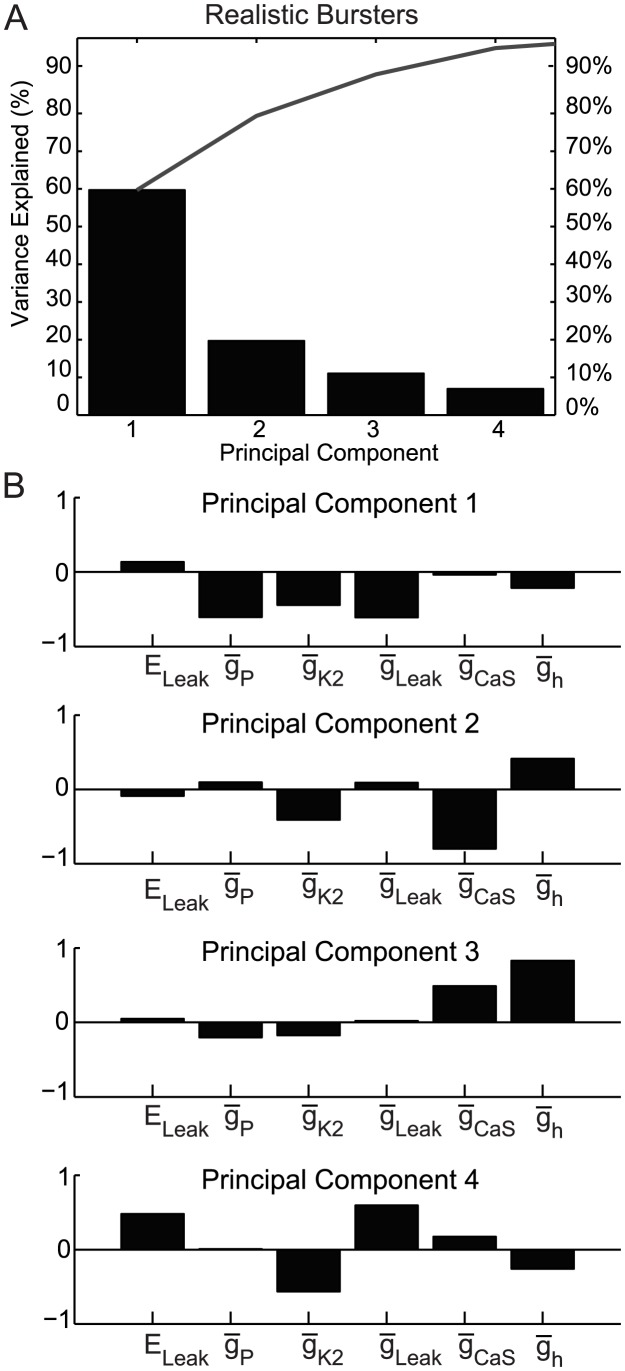
Principal components for the realistic bursters. (**A**) The most important principal components for the group are the first four components with their sum of variance >95%. The line above the bars shows the cumulative percentage of the variance. (**B**) The coefficients of the linear combinations of the parameters that generate these first four (principal) components.

To find interactions among the conductance parameters, we applied the Principal Component Analysis method (PCA) (see Methods) to each of our four groups of interest. For each group of interest, we plotted the percent of variability explained by each principal component (plots in panel A of Figures 5 and 6 for the realistic groups). Then, we identified the main principal components for each group as the smallest number of PCs for which the sum of their variances was greater than 95%. For each of these principal components we plotted the coefficients of their parameters (panel B of Figures 5 and 6). Tables S1A–D from Suppl. Material show the values of all the coefficients of the main principal components and figures S4A and S4B from Suppl. Material S4 show similar PC plots for the bursters and HCOs.

Figure 5A shows the PCA results for the realistic HCOs group. The first six principal components accounted for 96.6% of the variance. There was not much difference between the variance values of the main principal components (the biggest difference of 4.6% was between PC 4 and PC 5), which indicates that all main principal components have similar importance for this group. For the first four PCs, the coefficients found for the realistic HCO group (Figure 5) differed from those of the HCO group (figure not shown). For PC 5 and PC 6, these coefficients were quite similar for the two groups of instances, meaning that these linear combinations of conductances have some similar small influence on both groups. However, since there is no major differences in the amount of variance accounted for by each component, we could not discriminate one or two of these sets of coefficients as being the most influential in the realistic group's activity. Therefore, we hypothesize that there are no linear relationships between any sets of parameters that characterize this group's activity (same situation for HCOs).

We also applied PCA analysis to the groups of bursters (figure not shown) and realistic bursters (Figure 6). For both these groups the main principal components and their coefficients were remarkably similar. The first four (of the six total) components were main principal components that explained 96.88% and 97.32% respectively of the total variance. In both groups, there was a large difference between the amount of variance accounted for by the first and second components, which means the first component is the most important for these groups. The first component by itself explained 61.2% and 59.66% respectively, of the variance, which is very close to two-thirds of the total variance, so this component can be considered as sufficient to characterize the group or, for a more precise characterization, one can use the first three components, which together account for >90.3% of the variance. The coefficients of conductances that generate each of the first four PCs for both groups (Figure 6B) have the same sign (positive or negative) and only small differences in their values. In the first principal component, 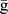
_P_, 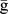
_K2_ and 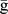
_Leak_ had large negative coefficients, while E_Leak_ was the only parameter with a positive coefficient albeit small. In the second PC, which accounted for only 17.3% and 19.7% of the variances respectively for these two groups, 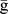
_CaS_ dominates followed by 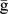
_K2_ with significant negative coefficients, while only 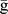
_h_ has a significant positive coefficient. However, since for both these groups the first PC is so large, we predicted that 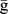
_P_, 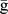
_K2_ and 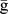
_Leak_, which dominate this component, should all show positive linear correlations in proportions to their weights (coefficients) and each should be negatively correlated with E_Leak_. Next, we explored visually these predictions of the PCA for our groups of interest.

### 5D clickable view for exploring relationships between parameters

We developed a Matlab tool to visualize five characteristics of a data set at once: in the present case three parameters which form a 3D parameter space of the data, the number of instances projected onto each point in this space given by the size of each point, and a fourth parameter which becomes visible when a point in this space is clicked with the mouse button. Each point clicked unveils a pie chart of the 4th parameter showing all instances projected onto this point in the 3D space. The pie chart is split into slices according to the number of values possible for the 4^th^ parameter: 5 slices if the 4^th^ parameter is E_Leak_ and in 8 slices each for the other parameters, with each slice having a different color. If there was no instance projected into the 3D space for a particular value of the 4^th^ parameter, then its corresponding slice was not shown in the pie chart. For a better visualization of the points projected onto the 3D plot, their deepness (i.e., their z- axis parameter values) was color coded with a colormap starting from dark blue shades for closest points (at 0% values) to light blue shades for farthest points (at 175% values). Each projected point was depicted by a circle filled with the color according to this mapping.

We used this 5D clickable tool to visualize the characteristics of our groups of interest. For each main principal component (see previous section) of each group, we selected its three parameters with the biggest coefficients that have the same sign (either positive or negative) and then we selected the parameter with the biggest coefficient of the opposite sign. The first 3 parameters selected were the parameters of the 3D space used by our clickable tool, and the last one was used for plotting the pie chart of each point plotted.

**Figure 7:**
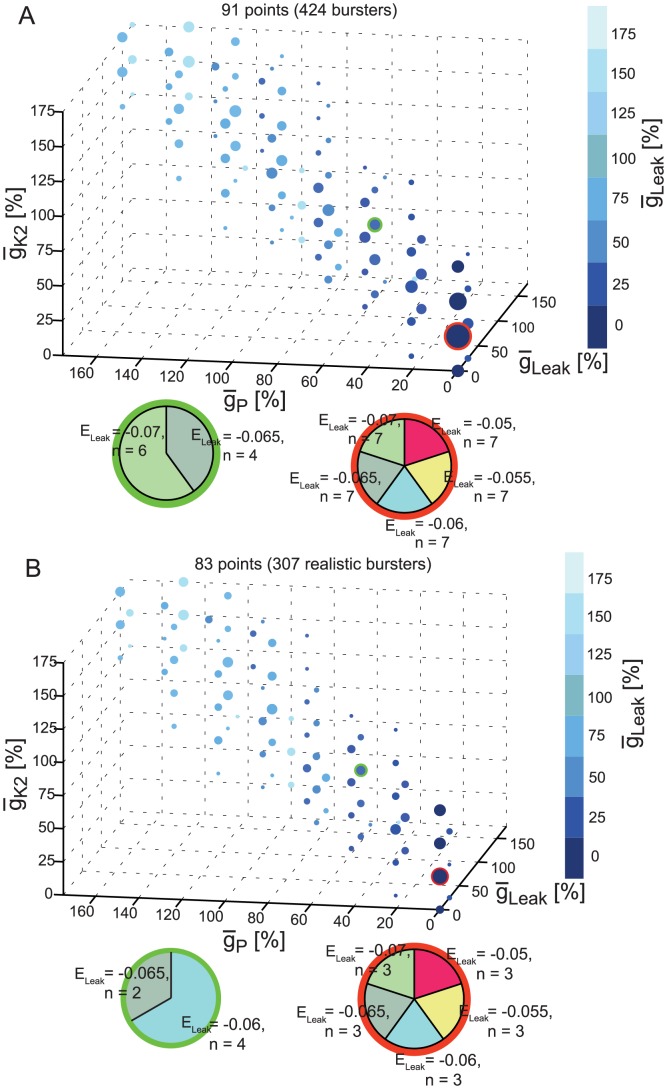
5D clickable view of the bursters and realistic bursters. Plot of all instances within a group projected onto the 3D space given by the maximal conductances of I_P_, I_K2_ and I_Leak._ The number of instances projected onto each point in the space is shown by the size of the circle surrounding it. Each point is clickable; it displays a pie chart of the leak reversal potential (E_Leak_) of all instances from the group having the same values of 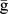
_P_, 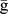
_K2_, and 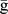
_Leak_ as the ones of the clicked point. Once clicked, a point (and its pie chart) are surrounded by a colored (here red and green) circle to differentiate it from the non-clicked points. (**A**) Burster instances. (**B**) Realistic burster instances.

Figure 7 shows the views obtained by applying our 5D clickable tool to the groups of bursters (Figure 7 A) and realistic bursters (Figure 7 B). In both groups, the first principal component was the most important PC for each group (with variances of approximately 60%). The three biggest coefficients of this PC were for 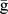
_P_, 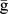
_Leak_, and 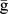
_K2_ (negative). We used them as the three axes of the 3D data projection space in our visualization tool. The biggest positive coefficient was for E_Leak_, which we used as the 4th parameter for display as pie charts in the visualization. The group of bursters had 91 points in the 3D space given by (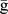
_P_, 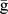
_K2_, 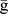
_Leak_), and the realistic group had 83 points in this space. In both groups, these points cluster around the main diagonal indicating that the amounts of 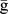
_P_, 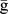
_K2_, and 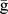
_Leak_ are positively correlated. Expansions of all the pie charts for both bursters and realistic bursters revealed that the range of permissible E_Leak_'s diminished and E_Leak_ had to be more negative as the values of 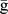
_P_, 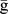
_K2_, 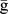
_Leak_ increased (from all E_Leak_ values for 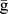
_P_  = 0 to only E_Leak_ = −0.07 for 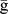
_P_ = 175%).

**Figure 8:**
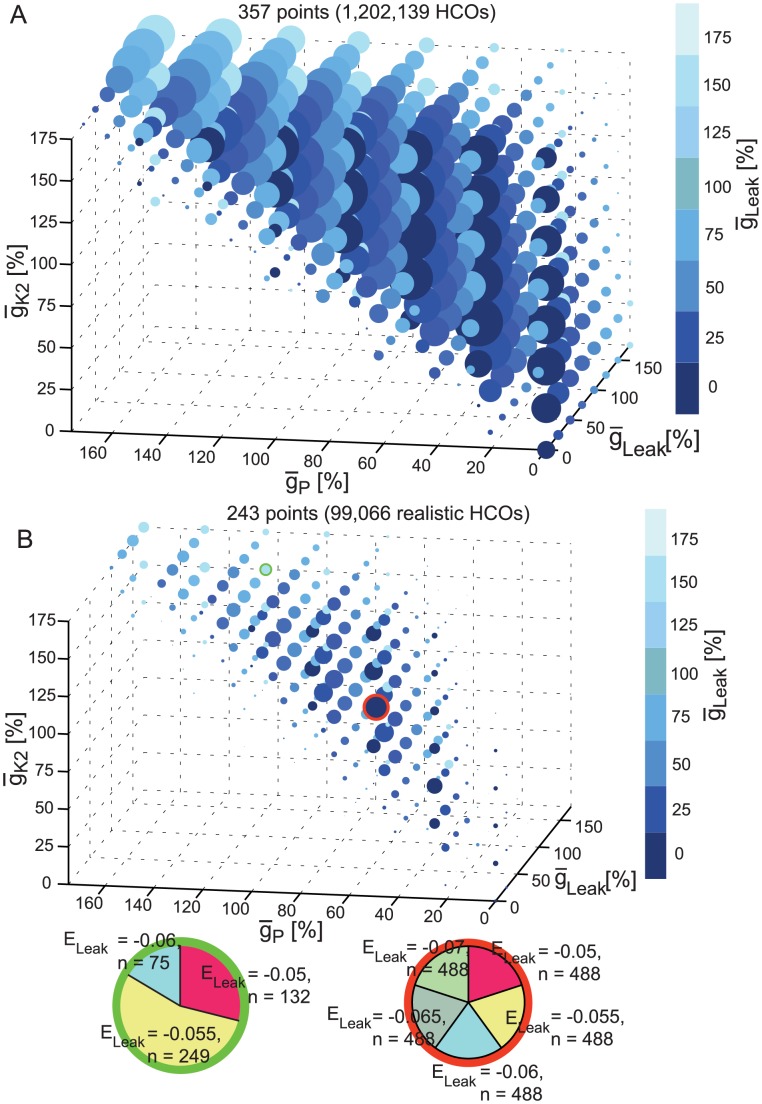
5D clickable view of the HCOs and realistic HCOs. (**A**) Plot of the HCO instances onto the 3D space given by the maximal conductances of I_P_, I_K2_ and I_Leak_. No clicked point is shown here. (**B**) Similar plot for the realistic HCO group. Each clicked point displays a pie chart of the E_Leak_ reversal potential of all instances from the group having the same values of 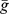
_P_, 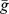
_K2_, and 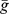
_Leak_ as the ones of the clicked point.

Figure 8 shows the views obtained by applying our 5D clickable tool to the groups of HCOs (Figure 8 A) and realistic (Figure 8 B) HCOs. In both groups, we used the 3D space given by 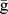
_P_, 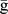
_K2_, and 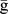
_Leak_. For the 4th parameter, we used E_Leak_. We chose this parameter space to compare the HCO groups with the burster groups in Figure 7. However, no clustering around the main diagonal, similar to that observed in Figure 7, emerged in these plots. Similar plots considering different parameters also failed to reveal such a relationship among parameters (see Supplemental Material Figure S3 for these plots). The plots in Figure 8 showed similar 3D shapes for the HCO and realistic HCO instances. This 3D shape has a complicated contour, similar to a wedge, and it is biased in the number of instances toward low 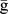
_Leak_. In contrast with burster instances (Figure 7) expansions of all the pie charts of Figure 8B revealed no contraction in the range of permissible E_Leak_'s until the highest (175%) values of 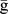
_P_, 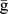
_K2_, 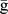
_Leak_ were reached, at which the E_Leak_‘s range was reduced to the four most negative values.

**Figure 9:**
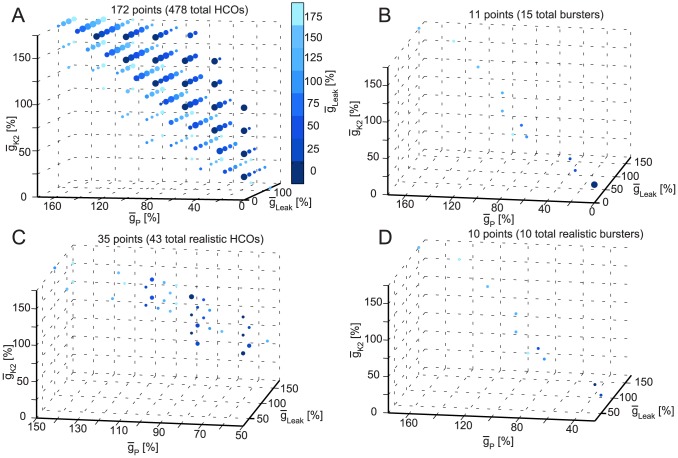
5D clickable views of the instances as projected onto the parameter space of 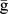
_Leak_, 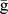
_K2_, and 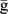
_P_ when maximal conductances of 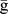
_CaS_, 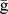
_h_, and of (if present) synapses are at the canonical values. (**A**) HCO instances. The plot shows a wedge-like 3D shape similar to the one in Figure 8A. (**B**) Burster instances with 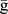
_CaS_ and 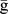
_h_ at the canonical values. (**C**) Realistic HCO instances. The cluster suggests linear correlations between the parameters. (**D**) Realistic burster instances with 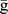
_CaS_ and 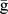
_h_ at the canonical value. Similar to burster instances case (Figure 9B), the plot shows instances situated close to a line marking the main diagonal of the space.

In Figure 9 we plotted the subset of instances from each group that have 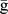
_CaS_, 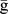
_h_, 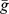
_SynS_, and 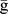
_SynG_ for the HCOs, and 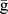
_CaS_ and 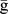
_h_ for the bursters at their canonical (100%) values (see Section Half-center oscillator model). For HCOs we had 478 such instances (Figure 9 A), for the realistic HCOs we had 43 instances (Figure 9 C), for the bursters we had 15 (Figure 9 B), and for the realistic bursters 10 (Figure 9 D). The plots of these points in the 3D space of (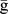
_P_, 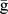
_K2_, 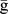
_Leak_) for burster groups (Figure 9 B and D) showed an apparent linear correlation. The subset of the realistic HCOs (Figure 9 C) also showed an apparent linear correlation between 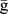
_Leak_, 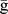
_P_, and 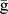
_K2_. Note that the boundaries of this cluster of points are defined by 50%–150% for 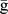
_P_, 25%–175% for 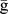
_Leak_, and 75%–175% for 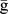
_K2_. These plots (Figure 9 B,C,D) show that, if for a group of instances one restricts the parameter space to an appropriate subset of parameters, then linear correlations between the remaining parameters may emerge. Next we investigated in more detail the apparent linear correlations observed between 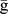
_P_, 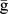
_K2_, and 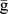
_Leak_ for the burster and realistic burster instances.

### 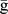
_P_, 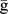
_K2_, and 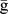
_Leak_ correlate linearly for the bursters and the realistic bursters

**Figure 10:**
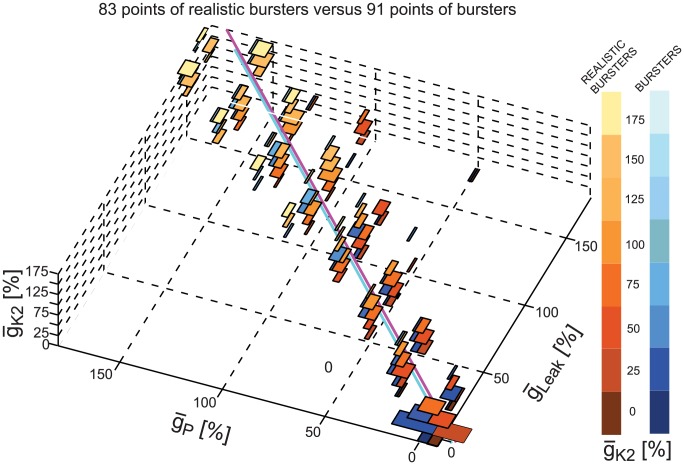
Comparison of the realistic and not realistic burster instances. Both groups show a linear correlation between maximal conductances of 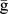 **_Leak_,** 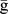 **_K2_, and** 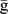 **_P_.** Instances from the realistic and not realistic burster groups are plotted in the space defined by 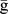
_P_, 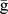
_K2_, 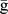
_Leak_. Burster instances that are not realistic are shown in blue shades and the realistic instances are shown in red shades. Values of 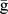
_K2_ are color coded from 0 with dark color to 175% with light color (color maps). The 3D orthogonal distance regression line corresponding to each group is shown with magenta for the realistic group and with cyan for not realistic subgroup.

Figure 10 shows a 3D plot of the instances of the burster groups into the space defined by 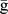
_P_, 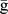
_K2_, 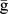
_Leak_. In this plot burster instances that are not realistic are shown in blue shades and the realistic burster instances are shown in red shades. Each point in this 3D space is depicted by a rectangle in (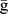
_P_, 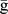
_Leak_) space. The number of instances projected onto each point in the space is shown by the size of a colored rectangle. Color maps show the value of 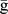
_K2_ going from 0 (dark shades) to 175% (light shades).

We fitted a least square fit regression line to each group of points (3D Orthogonal Distance Regression (ODR) line). The 3D ODR line is least distant from all the points and contains the centroid of points, which is the 3D point that has the mean of the points on the three axes. The direction vector that defines the line is given by the coefficients for the first principal component (PC), i.e., the first column of the coefficients returned by the PCA (in our case Matlab's princomp function) when applied to the set of 3D points (see Supplemental Material Text S1 for detailed mathematics and the coefficients of each equation corresponding to the two ODR lines shown in Figure 10, see [34] for Methods explanation, and [35] for an example).

For the realistic bursters the line is shown in Figure 10 in magenta, and for the not realistic bursters in cyan. The two lines did not intersect in the 3D space illustrated. However, their projections in either 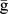
_Leak_, 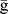
_K2_ plane or the 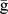
_P_, 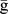
_K2_ plane did intersect with a small angle, but did not intersect in the 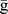
_Leak_, 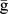
_P_ plane. That is, 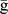
_Leak_ and 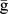
_K2_ (and similarly 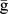
_P_ and 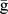
_K2_) have slightly different influences on the two groups of instances, while 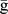
_Leak_ and 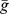
_P_ have the same effect on the two groups. Interestingly, the main diagonal of the 3D space of 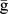
_Leak_, 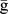
_P_, 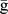
_K2_ passes though many points from either group. Both fitted lines are on the same side of the main diagonal (toward higher values of 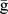
_Leak_), but do not intersect it in the space illustrated. These two lines seem almost parallel in the 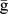
_Leak_, 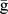
_P_ plane, with the magenta line further shifted toward higher values of 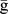
_Leak_ than the cyan line. The centroids and the two lines give insight into the characteristics of the two groups. The magenta line shows a tendency for the realistic instances to be at the high values on all axes. That is, large values of 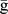
_P_, 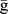
_K2_, and 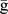
_Leak_ produced more realistic instances than the small values. The cyan line shows a tendency for the not realistic bursters to be at the low and middle values on all axes (in other words, small and moderate values of 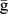
_P_, 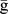
_K2_, and 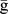
_Leak_ produced more not realistic instances than the large values).

The magenta line has a slightly less steep slope than the cyan line, and a moderate slope (with angle less than 45^o^) if compared with the main diagonal of the 3D space. Precisely, the starting point of the magenta line in the space shown is higher than the starting point of the cyan line, and the end point of the magenta line is lower than the end point of the cyan line. When there was no 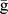
_P_ and 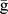
_Leak_, there were more realistic bursters for larger values of 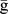
_K2_ and more not realistic bursters for the lower values of 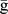
_K2_ (0%, 25%, and 50%). The larger the values of 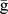
_P_, 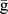
_K2_, and 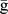
_Leak_ are, the larger the number of realistic instances and the smaller the number of the not realistic instances.

### Influence of 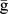
_P_, 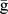
_K2_, and 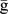
_Leak_ on the realistic instances

**Figure 11:**
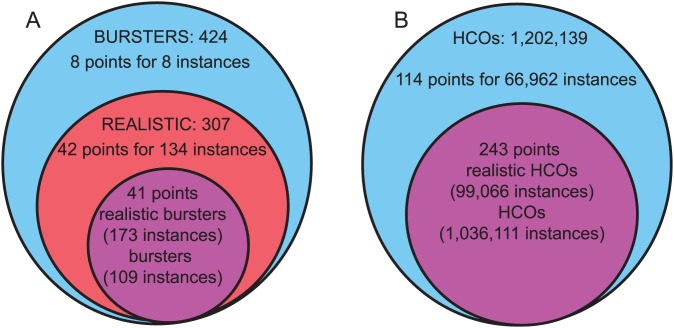
Venn diagram of the distribution of instances projected in the 3D space defined by 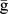
_Leak_, 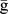
K2, and 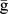
_P_. (**A**) Bursters and realistic bursters. The sets showing the number of points in the 3D space (shaded rectangles of Figure 10) that include only not realistic bursters (8), only realistic bursters (42), and both types of instances (41) are colored in light blue, red, and purple, respectively. (**B**) HCOs and realistic HCO instances. The sets showing the number of points in the same 3D space as Figure 8 (data not shown) that include only not realistic HCOs (114), and both types of instances (243), not realistic and realistic, are colored in blue, and purple, respectively.

We explored the overlap of not realistic and realistic bursters in the 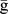
_P_, 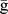
_K2_, and 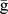
_Leak_ space of Figure 10. Some of the points (shaded rectangles) in this linear relationship (50 out of the total of 91) represent only not realistic or only realistic instances. Eight points (light blue circle), each corresponding to eight instances, were characterized as only not realistic (Figure 11 A). They were separated from the 42 points (red circle), corresponding to 134 instances, that were characterized as only realistic. However, 41 points (purple circle) represented 282 both realistic and not realistic instances. Thus there are a total of 424 instances represented in Figure 10. Having both realistic and not realistic burster instances projected into the same point in (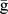
_P_, 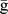
_K2_, 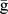
_Leak_) space means that these instances were influenced by additional parameters (either 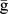
_CaS_, 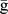
_h_, or E_Leak_) toward being realistic or not.

We also explored overlap for the HCOs in in the same 3D space as Figure 10 (data not shown) and found that the three maximal conductances of 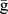
_P_, 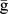
_K2_, and 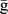
_Leak_ were not sufficient to characterize uniquely realistic instances (Figure 11 B). 114 points (light blue circle), corresponding to 66,962 instances, were characterized as not realistic HCOs (i.e., they are HCO instances that do not satisfy the necessary criteria to be also characterized as realistic instances). 243 points (purple circle) included both realistic and not realistic HCO instances. Having both realistic and not realistic HCO instances projected into the same point in (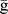
_P_, 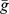
_K2_, 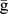
_Leak_) space suggests that additional parameters are needed to separate these two types of instances (either 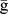
_CaS_, 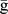
_h_, 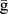
_SynS_, 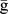
_SynG_, or E_Leak_). Thus, for HCOs there is no point in the 3D space that represents only realistic instances (Figure 11 B), which means that these instances are characterized by more than the parameters which define the 3D space.

**Figure 12:**
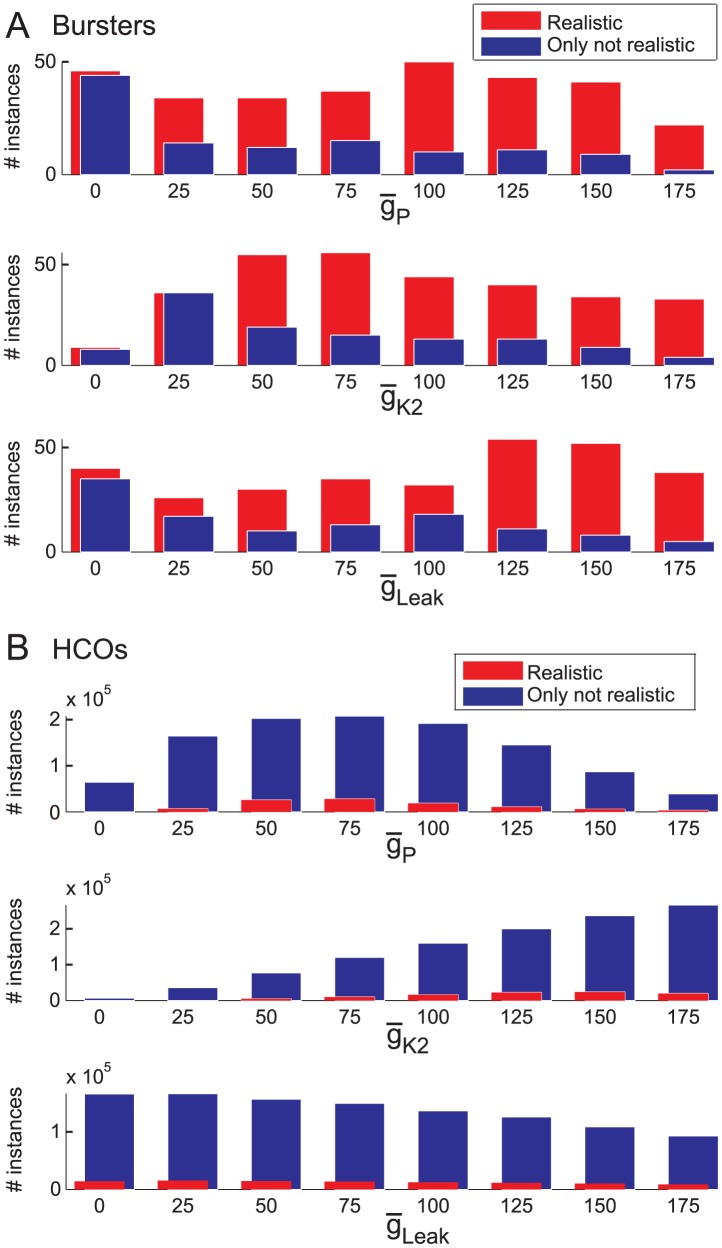
Histograms showing the distributions of instances projected onto the three axes of 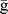
_Leak_, 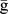
_K2_, and 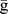
_P_. The histogram corresponding to one group of instances was shifted for visualization of the overlapping bins. The two-sample Kolmogorov-Smirnov test rejected the null hypothesis (that the two distributions are from the same continuous distribution) at the 5% significance level for each axis (h = 1 in all cases). (**A**) Distributions of the realistic and not realistic burster instances. (**B**) Distributions of the realistic and not realistic HCO instances.

We then analyzed how the three parameters (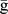
_P_, 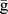
_K2_, 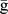
_Leak_) were distributed in the groups of bursters and HCOs. Figure 12 A shows the distributions of the realistic and of the not realistic bursters on each of the three axes of the 3D space given by 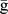
_P_, 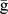
_K2_, and 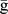
_Leak_ (Figure 10). We performed a two-sample Kolmogorov-Smirnov test on the two distributions obtained on each axis. Each test rejected the null hypothesis that the two distributions were from the same continuous distribution at the 5% significance level (h = 1 in all cases, p_Leak_ = 0.0014, k_Leak_ = 0.875, p_P_  = 0.0014, k_P_  = 0.875, p_K2_  = 0.0098, k_K2_ = 0.75). Having different distributions for the two groups indicates that the realistic instances showed parameter distributions which can be used to separate them from the not realistic instances. First, both groups show almost the same number of instances if any of 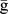
_P_, 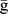
_K2_, or 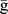
_Leak_ is equal to 0. Then, the distributions diverge. On the 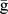
_P_ axis, the number of not realistic instances decreased and stayed below 18 with an increase in 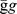
_P_ whereas for 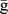
_P_ = 0 both groups have a large number of instances. The distribution of the realistic instances on the 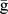
_P_ axis, showed no clear tendency with a decrease in the number of instances for 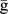
_P_ = 25% followed by an increase at 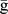
_P_ = 100% then again a decrease with a minimum at 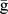
_P_ = 175%. On the 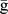
_Leak_ axis, the two distributions seem to have the same tendencies as the distributions on the 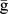
_P_ axis, but here the peak in number of realistic instances occurred for 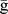
_Leak_ = 125%. On the 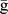
_K2_ axis, the distribution of the not realistic bursters showed a peak for 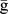
_K2_ = 25%, then the distribution showed a continuous decrease in the number of instances with increasing 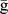
_K2_. On this axis, the realistic distribution had a peak at 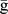
_K2_ = 75%, followed by a slight decrease with increasing 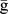
_K2_. Summing up, the largest number of only not realistic bursters occurred for small values of these conductances (0–25%) and the largest number of realistic bursters occurred for moderate to large values of these conductances (50–150%).

Figure 12 B shows the distributions of the realistic and not realistic HCO instances on each of the three axes of the 3D space given by 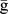
_P_, 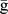
_K2_, and 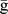
_Leak_. Similar to burster instances shown above, the two-sample Kolmogorov-Smirnov test applied to these two distributions rejected the null hypothesis (p>0.05) that they are from the same continuous distribution (h = 1 in all cases, p_Leak_ = 0.00015562, k_Leak_ = 1, p_P_  = 0.00015562, k_P_  = 1, p_K2_  = 0.0014, k_K2_  = 0.875). On the 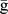
_P_ axis, both distributions showed a peak in their number of instances at 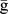
_P_ = 75%. On the 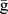
_K2_ axis, the distribution of the not realistic HCO instances showed a continuous increase in number of instances with increasing 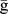
_K2_. The distribution of the realistic HCO instances showed a peak in the number of instances at 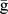
_K2_ = 150%. On the 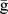
_Leak_ axis, the distribution of the not realistic HCO instances showed a continuous decrease in number of instances with increasing 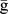
_Leak_. The distribution of the realistic HCO instances showed a peak in the number of instances at 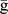
_K2_ = 25%, then a continuous decrease in the number of instances with increasing 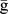
_Leak_. Summing up, the largest number of realistic HCO instances occurred for moderate values of 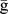
_P_ (50–125%) and large values of 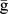
_K2_ (canonical and above, 100–175%). 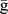
_Leak_ above 125% reduced the number of realistic HCO instances.

### Period's sensitivity to variations of 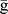
's

To assess the sensitivity of period to variation of 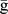
_Leak_, 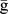
_K2,_ and 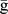
_P_, we queried the HCO-db database to build up the families existing within each group of interest. We define a **family** as being a sub-set (of a group) of instances that have all the same parameter values except one (e.g., all realistic bursters that vary only by 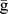
_P_ constitute a family). Note that one can partition a group into families according to the number of members in the family. Surprisingly, for the groups of bursters and realistic bursters, all the instances of each group were part of families of one member only when 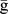
_P_ was varied, which means that activity of these groups of instances is very sensitive (i.e. not robust) to changes in 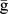
_P_ and the effect of 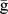
_P_ on period could not be assessed. The activity of the realistic bursters group also turned out to be very sensitive also to changes in 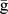
_Leak_, with all instances being part of families of only one member. The activity of the bursters was slightly less sensitive to changes in 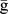
_Leak_ with 423 instances as members of families of one member and just 2 instances as members of a family of 2 members, which also precluded an assessment of an effect 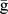
_Leak_ on period. From previous research [3], [11], [36], we knew that all three of these parameters influence the activity type of model instances. However, while we did expect the activity of the burster instances to be very sensitive to 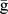
_Leak_ [3], we did not expect such a big influence of 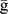
_P_. Finally, the activity of the realistic bursters showed less sensitivity to 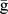
_K2_ variations than to 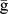
_Leak_ and 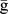
_P_ variations including 26 families of 2 members and 2 families of 3 members. Burster instances showed similar sensitivity to changes in 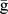
_K2_ variations as the realistic instances including families of 2, 3, and 4 members (45, 2, and 1, respectively). For all families of both bursters and realistic bursters, an increase in 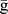
_K2_ resulted in a monotonic decrease of the period. For each family, first we ordered in ascending order its members by the amount of the parameter varied (here 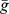
_K2_), and then we calculated the variation (here decrease) of the period as being the difference between the periods of the last and first members of the respective family. The average decrease of the period for each family were: −2.43 s (range −4.46 to −0.52) for realistic instances of families of 2 members; −3.16 s (range −3.18 to −3.14) for realistic instances of families of 3 members; −2.41 s (range −4.46 to −0.52) for burster instances of families of 2 members; −3.35 s (range −3.52 to −3.18) for bursters of families of 3 members; and the only family of 4 members of the bursters had a decrease of −4.18 s of the period values.

The activity of the realistic HCO group was quite sensitive to 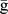
_P_ also (92,970 families of one member, and 3,048 families of 2 members), but less sensitive than for the realistic burster instances. As expected [3], the activity of this group was less sensitive to 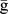
_Leak_ than the activities of the burster and realistic burster groups (66,611; 11,873; 2133; 452; 92; and 7, respectively, families of 1–6 members). Finally, the activity of the realistic HCO group showed a lesser sensitivity to changes in 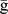
_K2_ than the sensitivity to 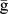
_Leak_ (68,702 families of one member, 13,163 families of 2 members, 1,299 families of 3 members, 34 families of 4, and 1 family of 5 members). As found by Hill et al. [29], an increase in 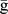
_K2_ resulted in a monotonic decrease of the period for most of the families of this group. Only 19 (out of 13,163) families of 2 members showed an increase in period. The average decrease of the period for each family were: −3.32 s (range −9.75 to 1.29) for families of 2 members; −4.87 s (range −9.65 to −0.38) for families of 3 members; −6.59 s (range −9.09 to −2.31) for families of 4 members; and the only family of 5 members had a decrease of −9.46 s of the period values. Then, an increase of 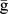
_P_ resulted in an increase of the period for most 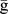
_P_ families of 2 members (3,014 out of 3,048). The average increase in the period values was 4.255 s (range −1.507 to 9.778) for all these 2 member families. Based on the large number of multimember families, it seems that in the heartbeat HCO, inhibition changes the influence of 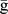
_P_, 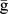
_K2_, and 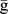
_Leak_ on network activity by making the activity of HCO instances more robust to changes in these parameters.

## Discussion

How does a given aspect of the electrical activity of neurons and networks change as the value of a parameter changes? Here, we focused on how intrinsic membrane and synaptic parameters interact to maintain functional bursting activity in an HCO model and in bursters from this HCO. Specifically, we asked how does the bursting activity of leech heart interneurons in isolation or in an HCO change by changing parameters in a defined parameter space? To systematically explore the parameter space of the HCO and corresponding burster models, in our previous work [31], we simulated about 10.5 million model instances, whose characteristics we recorded into a database named HCO-db [31], [32].

We tried dimensional stacking and other techniques [25], [26], [37] to visualize globally the effect of parameter changes on activity type, but it was difficult to see the effects of all parameters. So we simplified by looking at the influence of only two parameters at a time (see Results, sub-section Visualizing interactions in pairwise parameter variations).

### Pairwise correlations in model isolated HN neurons and HN HCOs

Similar to the results presented in [3], our pairwise plots revealed that several parameters work in pairs to produce more burster (figure not shown) and realistic burster instances (Figure 4): increasing 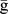
_h_ together with more hyperpolarized E_Leak_; making 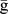
_CaS_ larger, together with a larger 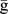
_Leak_ (not monotonically, but with a wave shape); increasing 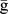
_K2_ together with more depolarized E_Leak_; increasing 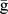
_Leak_ (above 50%) together with more hyperpolarized E_Leak_ - for more depolarized E_Leak_ and smaller 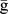
_Leak_ there were less number of instances in each group than when E_Leak_ was more hyperpolarized. For the HCOs (figure not shown) and realistic HCOs (Figure 3), decreasing 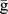
_Leak_ together with more depolarized E_Leak_ produced the most instances (maximum at 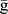
_Leak_ = 25%). However, from our pairwise plots we cannot assess the potential relationships between three parameters, as for example those stated in [3] between 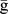
_Leak_, E_Leak_ and 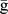
_CaS_. Our aim here was to find all potential existing correlations in our models whether between two, three or more parameters.

### Linear correlation between 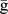
_Leak_, 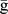
_K2,_ and 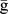
_P_ characterizes realistic bursting in isolated neuron instances (bursters)

Recent studies, both experimental [16], [17], [19], [23], [27] and modeling [11], [21], [20], [38], have shown in several systems that consistent activity is maintained despite a 3–5 fold variation from animal to animal or model instance to model instance among ionic and synaptic conductances and that correlations exist between these parameters. Thus the suggestion has arisen that the functional activity of a given neuron may reside in the set of parameter correlation rules it maintains rather than in the value of any particular parameter. However, the precise combinations of parameters that is adequate to preserve functional neuronal or network activity in not fully elucidated for any system. Most studies have reported positive linear correlations [19], [23], while recently [21], [27] have shown negative linear correlations, and several theoretical studies have reported the existence of nonlinear correlations [11], [20]. Such correlations have been reported in single cells [39] and in networks of two or more cells correlations [19], [27] and they have been found between two parameters [21], [27], as well as three or four parameters [3], [27], [40], [17]. The potential for general insights into mechanisms for bursting in single neurons and HCOs motivated us to pursue this modeling study despite challenges imposed by large 8-dimensional parameter space that it presented.

To find potential linear correlations among our varied parameters, we applied PCA to our four activity groups of interest. This method showed that for the bursters and the realistic bursters groups there is a linear correlation between the six parameters out of which 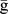
_Leak_, 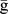
_K2,_ and 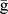
_P_ had each about 3 times or more importance than the other three parameters (Figure 6). Plots (Figure 7 A, B) of each group's instances in the 3D space of 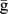
_Leak_, 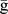
_K2,_ and 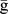
_P_ showed a linear correlation for each group around the main diagonal (Figure 10). Two corollaries emerged from our analysis: 1) for the bursters and the realistic bursters the range of permissible E_Leak_'s diminishes and E_Leak_ must be more negative as the values of 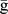
_Leak_, 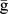
_K2,_ and 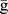
_P_ increase (Figure 7); and 2) moderate to large values of 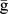
_Leak_, 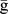
_K2,_ and 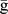
_P_ produced more realistic bursters than the small values, and small to moderate values of 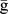
_Leak_, 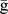
_K2_, and 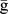
_P_ produced more not realistic bursters than the large values (Figure 12).

The above observations indicate that these three conductances work together to produce burster and realistic burster instances and begin to pinpoint the mechanisms supporting bursting in isolated heart (HN) interneurons. Olypher and Calabrese [11] used sensitivity analyses to predict coordinated changes of parameters that would lead to constant activity in the HN HCO model. They found that 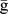
_P_ opposes both 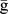
_Leak_ and 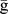
_K2_, with 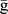
_Leak_ and 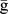
_K2_ having negative relative sensitivity of almost half of 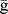
_P_‘s relative sensitivity (positive). Our analyses (Figures 7 and 10) yield similar yet contrasting results; 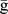
_Leak_ opposes 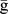
_P_, but in an almost equal relationship (see their weights in Figure 6 B). A small amount of 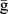
_Leak_ requires a small amount of 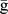
_P_ to produce bursting, and a large amount of 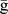
_Leak_ requires a large amount of 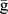
_P_ (within some range). In addition, if 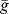
_P_ is small then 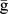
_K2_ is small (within a range), and if 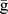
_P_ is large then 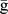
_K2_ must be large. Interestingly, all three parameters have negative weights in the equation given by the PCA method, with 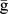
_P_ and 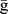
_Leak_ having almost equal weights and being slightly bigger than the weight of 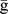
_K2._ Thus it appears that none of these three parameters is sufficient by itself to produce burster and realistic burster instances, but they must work together (in linear combination) in almost equal amounts towards producing the respective instances. PCA guarantees that all existing linear combinations (in any number of parameters) that characterize a set of data will be found, should such linear combinations exists. Thus our bursters and realistic bursters groups are characterized by the single dominant linear combination between parameters returned by the PCA method (Figure 6 A).

From a mechanistic standpoint, the observed correlation of 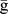
_Leak_, 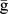
_K2,_ and 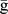
_P_ and their corollaries fit our current understanding of bursting in HN neurons. None of these three currents show inactivation and their activation is relatively fast compared to the burst period (instantaneous in the case of I_Leak_). I_K2_ is active only during the burst phase, owing to its depolarized range of activation, and provides outward current that limits depolarization. I_P_ is active throughout the burst cycle owing to it broad and shallow activation curve, and it provides the inward current that drives baseline spiking activity. I_Leak_ is also active throughout the burst cycle and provides the outward current necessary for repolarization after the burst. Essentially I_K2_ must oppose I_P_ during the burst and I_Leak_ must oppose it during the interburst interval. When 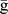
_Leak_, 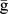
_K2,_ and 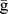
_P_ are all small then I_P_ is very weak during the interburst interval and a small I_Leak_ (i.e., small 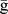
_Leak_) even with a relatively depolarized E_Leak_ can effectively oppose it. But when 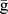
_P_ is moderate or large (i.e., a large I_P_) then a large 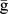
_Leak_ with a relatively negative E_Leak_ (i.e., a large I_Leak_) is necessary to oppose I_P_ during the interburst interval (Figure 7 pie charts). Based on work with canonical HN models and analyses in the living neurons [29], [3], [13] we hypothesize that I_CaS_ is critical for bursting in isolated HN neurons and that the burst duration is controlled by its inactivation dynamics, which in our model are fixed. This hypothesis is further supported by the data of Figure 4 C. Because I_Leak_ is the main determinant of the interburst interval, a small 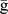
_Leak_ will then lead to short interburst intervals and thus to more not realistic burster instances (Figure 12).

### An absence of linear correlations between parameters characterizes HCO and realistic HCO instances

For the groups of realistic and HCO instances, PCA did not find any linear relationship between the parameters (Figure 5). Plots (Figure 8 A, B) of the instances of each HCO group in the 3D space defined by 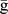
_Leak_, 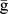
_K2,_ and 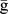
_P_, which revealed correlations for the burster groups, showed that these groups of instances form complex shape, like a wedge. Goldman et al. [7] in their study on the robustness of activity type in single model neurons found similar relationships between parameters supporting bursting. This non-linearity suggests that these three parameters are not enough to characterize the HCO and realistic HCO groups. We hypothesize that 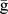
_Leak_, 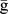
_K2,_ and 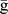
_P_ play the same role in HCOs as outlined above for bursters: the added factor being synaptic inhibition, which provides outward current during the interburst interval. Because synaptic inhibition provides outward current during the interburst interval, the system no longer depends on a large 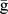
_Leak_ with a relatively negative E_Leak_ to oppose a large I_P_, and in fact small 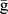
_Leak_'s are favored (Figure 8). Note that the pie chart analysis of Figure 8 B shows directly a lack of restriction on E_Leak_ throughout the region of realistic HCO bursting. Figure 3 further corroborate this hypothesis by showing that the number of realistic HCO instances increases dramatically as 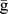
_SynS_ increases.

Our plots in Figure 9 show that one can obtain linear correlations between parameters for HCOs when working in a reduced parameter space. In our case, we reduced the parameter space to only three maximal conductances, 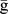
_Leak_, 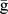
_K2,_ and 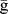
_P_, and kept the rest of the parameters at their canonical values. Then we plotted the instances of our four groups of interests into this new 3D space. The plot of the reduced set of HCO instances still showed a complex relationship between the three parameters, while the plot of the reduced sets of realistic HCO instances (and also of bursters and realistic bursters instances) showed linearity. The reduced set of realistic HCOs produced a linear cluster in this space, and the reduced sets of bursters and realistic bursters produced lines similar to those seen to the unreduced sets (Figure 10).

### How robust are the realistic bursters to variation of 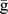
_Leak_, 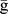
_K2,_ and 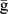
_P_?

Our analysis here indicates that a strong correlation between 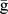
_Leak_, 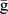
_K2,_ and 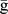
_P_, is critical in determining activity in bursters and realistic bursters and thus bursting activity should be very sensitive to their individual variation. Sensitivity analysis is the most common computational method used to assess the influence of a parameter on the activity type in neuronal models [29], [11], [7], [36]. Previous work showed that the bursting activity of isolated HN model neurons is very sensitive to 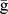
_Leak_, so that HN neurons are not robust busters under experimental conditions that alter leak properties [3]. We have confirmed and extended that finding here using our database of model instances by showing that 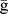
_Leak_ families have only one member. We have similarly shown a strong sensitivity to 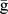
_P_ and to a slightly lesser extent to 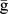
_K2_. Robustness of activity state requires correlated changes in these three parameters.

When configured as an HCO, realistic bursting activity becomes substantially more robust to individual changes in these parameters, which can be seen as both the expanded occupancy of the parameter space in Figure 8 and by the large number of 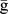
_Leak_, 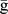
_K2,_ and 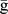
_P_ families with multiple members. Thus mutual synaptic inhibition adds robustness to bursting activity in HN neurons.

### Correlated conductance and robustness of activity states

Several recent studies suggest that correlated parameters could be key factors in maintaining functional activity states in neurons. Goldman et al. [7] found that a model neuron's robustness (ability to maintain functional activity, e.g. regular bursting) is determined by its sensitivity to sets of parameter changes. Hudson and Prinz [21] found that conductance correlations contribute to the robustness of critical features of electrical activity. Lamb and Calabrese [41] found partial conductance correlations that contribute to the activity phase of the leech heart motor neurons. The mechanisms that maintain functional bursting in the pyloric CPG of the stomatogastric nervous system of crabs employ several key parameter correlations (linearly or not) [17], [18], [20], [21], [23] which appear necessary for the maintenance of activity state. Likely evolution promoted such mechanisms to maintain robust activity and robustness seems to be achieved in the oscillator heart interneurons of the leech heartbeat CPG by three linearly correlated maximal conductances of 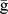
_P_, 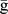
_Leak_ and 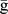
_K2._ Changes in any of these three parameters (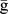
_P_, 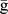
_Leak_ or 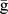
_K2_) must be accompanied by changes in the other two parameters in a linear correlation to maintain the neurons in a realistic bursting activity mode. Our results imply that these three parameters compensate for each other's variations to keep bursting functional. Moreover they show that linking these neurons by mutually inhibitory synapses into a HCO increases robustness. We leave unanswered for future work the question of how period is modulated while robustness is maintained.

## Methods

### Half- center oscillator model (HCO)

We used Hill et al.'s model [29] of a half-center oscillator (HCO) which produces electrical activity (rhythmic alternating bursting of mutually inhibitory neurons) similar to that observed in the living system (in the heartbeat central pattern generator or CPG of the leech). The model is publicly available on ModelDB repository (https://senselab.med.yale.edu/ModelDB/), accession number 19698. The HCO model consists of a two reciprocally inhibitory model interneurons, represented as single isopotential electrical compartments with Hodgkin and Huxley [30] type intrinsic and synaptic membrane conductances. Each compartment contains 8 voltage-dependent currents, five inward currents I_Na_ - a fast Na^+^ current, I_P_ - a persistent Na^+^ current, I_CaF_ - a rapidly inactivating low-threshold Ca current, I_CaS_ - a slowly inactivating low-threshold Ca current, I_h_ - a hyperpolarization-activated cation current) and three outward currents (I_K1_ - a delayed rectifier-like K current, I_K2_ - a persistent K current, I_KA_ - a fast transient K current). The model has two types of inhibitory synaptic transmission between the two interneurons: graded transmission (SynG) and spike-mediated transmission (SynS). The graded transmission SynG was modeled as a postsynaptic conductance controlled by presynaptic Ca^2+^ concentration and the spike-mediated transmission SynS was modeled as a postsynaptic conductance triggered by presynaptic spikes. The values for the maximal conductances and the leak reversal potential (free parameters in the model) that we used for our canonical model are 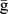
_CaS_ = 3.2 nS, 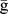
_h_ = 4 nS, 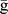
_P_ = 7 nS, 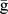
_K2_ = 80 nS, 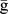
_Leak_ = 8 nS, 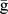
_SynS_ = 60 nS, 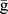
_SynG_ = 30 nS, 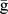
_Na_ = 200 nS, 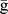
_CaF_ = 5 nS, 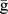
_K1_ = 100 nS, 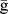
_KA_ = 80 nS, and E_Leak_ = −60 mV [31]. The kinetics, voltage-dependencies, reversal potentials of the intrinsic currents, and the synaptic connections of the HCO model interneurons have all been verified and previously adjusted to fit the biological data of leech interneurons [2], [14], [29], [36], [42]. The differential equations of the model are given in the following.

The equation of the membrane potential (V) of each neuron is given by:
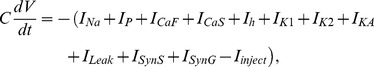
where C is the total membrane capacitance (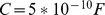), I_ion_ is an intrinsic voltage-gated current, I_Leak_ is the leak current, I_SynS_ is the graded synaptic current, I_SynS_ is the spike-mediated synaptic current, and I_inject_ is the injected current. Voltage-gated currents are given by



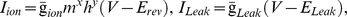


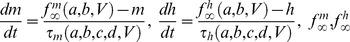





 given by 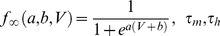




 given by 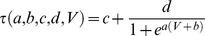 where 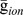 is the maximal conductance, E_ion_ is the reversal potential, and the activation and inactivation variables for the parameters are given in Table 1.

**Table 1:** Voltage-dependent activation (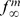), inactivation (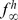), time constant of activation (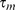), and time constant of inactivation (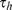) of the ionic currents used in the Half-center Oscillator Model ([29]).

	x	y	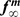		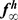		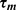				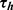			
			a	b	a	b	A	b	c	d	a	b	c	d
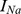	3	1	−150	0.029	500	0.03	**				**			
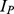	1	0	−120	0.039			400	0.057	0.01	0.2				
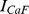	2	1	−600	0.0467	350	0.0555	***				270	0.055	0.06	0.31
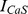	2	1	−420	0.0472	360	0.055	−400	0.0487	0.005	0.134	−250	0.043	0.2	5.25
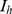	2	0	*				−100	0.073	0.7	1.7				
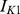	2	1	−143	0.021	111	0.028	150	0.016	0.001	0.011	−143	0.013	0.5	0.2
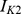	2	0	−83	0.02			200	0.035	0.057	0.043				
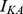	2	1	−130	0.044	160	0.063	200	0.03	0.005	0.011	−300	0.055	0.026	0.0085

* The steady-state activation of I_h_ is given by 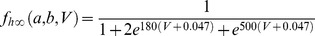.

**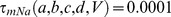, 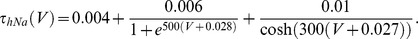

***
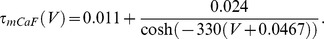



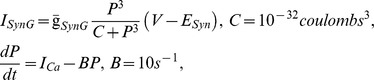


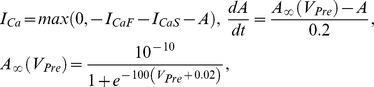
where 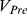 is the presynaptic membrane potential.
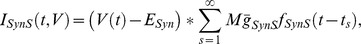
where 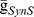 is the maximal synaptic conductance, 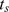 is the time of a spike event, and *M* is the modulation variable of the synapse determined from 
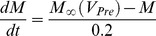
, 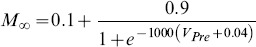. The synaptic function is given by 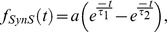
where 
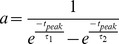
 is a normalization constant with 
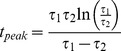
, and the decay (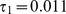) and rise (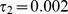) times of the synaptic conductance.

In our previous work [31], we performed extensive simulations of this HCO model by systematically varying eight key parameters (a brute-force approach). Figures 2 A and B show the intrinsic currents and the synaptic conductances of two randomly chosen simulated instances, one a realistic HCO and one a realistic burster. The figures show how the currents interact (including their ratio and the timing) to produce the realistic leech bursting activity. One can see in the examples that I_A_ and I_CaF_ are very small, and thus have a small influence on the cell's activity. I_Na_ and I_K1_ are mainly involved is spiking and must be co-varied to maintain constant spiking during the burst. While spike frequency is an important determinant of HCO activity due to inhibition of the opposite neuron, it is better controlled by slow currents and spike amplitude and undershoot do not appear critical for model behavior. We simply came upon a canonical combination for these two currents that produced realistically sized spikes. Moreover, previous analysis involving varying one parameter at a time had identified maximal conductances 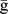
_SynS_, 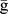
_SynG_, 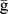
_P_, 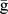
_K2_, 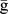
_h_, 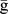
_CaS_, and 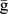
_Leak_, and E_Leak_ as critically contributing to bursting behavior [3]. Thus in this analysis we did not vary 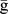
_K1_, 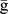
_KA_, 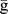
_CaS_, or 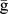
_Na_ but concentrated on varying the critical parameters. As some selection was necessary due to computational and financial limitations on database size, this selection seemed reasonable. In future we may be able to add the fast currents to the database.

All model simulations were started from the same initial conditions, which were different for each of the two neurons and were obtained by running the canonical HCO model [29] for 200 s, such that one of the two neurons was in its bursting state and the other one was being inhibited. The same parameter values were used in each of the paired model neurons. The eight parameters varied were: the maximal conductances of spike-mediated (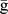
_SynS_), graded transmission (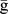
_SynG_), 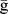
_Leak_, 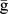
_P_, 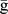
_CaS_, 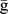
_h_, and 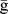
_K2_, across of 0%, 25%, 50%, 100%, 125%, 150%, and 175% of their canonical values and E_Leak_ across −70, −65, −60, −55, and −50 mV values. After changing a parameter, we ran each model instance for 100 s to allow the system to establish stable activity, and then we ran it for another 100 s, from which we recorded the voltage traces of the electrical activity corresponding to its paired neurons and the corresponding spike times. The firing characteristics were analyzed and recorded into a database named HCO-db.

### Definitions

In voltage traces we recognized a **spike** only if the potential waveform crossed a threshold of −20 mV. We defined a **burst** as having at least three spikes and a minimum inter-burst interval of 1 second. We defined the **cycle period** as being the interval between the middle spikes of two consecutive bursts. **Phase** was calculated on a per cycle basis, as being the delay from the middle spike of a burst of neuron B to the middle spike of the preceding burst of neuron A divided by the interval from this middle spike of the next burst of neuron A to the middle spike of the preceding burst of neuron A. The **duty cycle** was defined as the percentage of the period occupied by a burst.

We defined a **half-center oscillator** instance (**HCO**) as having: two model interneurons each showing bursting activity with at least two bursts in a 40 s time interval, with each burst having normal spikes (coefficient of variation of the amplitudes of the spikes within any burst is less than 0.07); a small variation of period (coefficient of variation of period less than 0.05); relative phase in the range of (0.45–0.55); and at least one synaptic component present (either 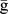
_SynS_≠0, or 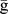
_SynG_≠0, or both 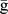
_SynS_≠0 and 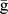
_SynG_≠0). We considered a **realistic HCO** instance as being a HCO that showed realistic bursting corresponding to that observed in leech oscillator heart interneurons. Precisely, it was a HCO with period between 5–15 s, average spike frequency between 8–25 Hz, and duty cycle between 50–70%.

We defined an **isolated neuron** instance (**isolated neuron**) as having two identical interneurons (though started with different initial conditions, but otherwise identical), and no synaptic interaction (i.e., 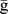
_SynS_ = 0 and 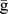
_SynG_ = 0). We defined a **burster** instance as being an isolated neuron instance for which both neurons had at least two bursts, each with normal spikes, and regular periods (as defined above for the HCOs). Note that burster instances can be thought of as being HCOs with no synaptic connections. We defined a **realistic burster** as being a burster that showed realistic bursting corresponding to isolated leech oscillator heart interneurons. Precisely, it was a burster with period between 5–15 s, and average spike frequency between 8–25 Hz. Note that realistic bursters can be thought of as being realistic HCOs with no synaptic connections. We define a **family** as being a sub-set (of a group) of instances that have all the same parameter values except one (e.g., all realistic bursters that vary only by 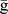
_P_ constitute a family). Note that one can partition a group into families according to the number of members in the family.

### HCO database

In our previous work [31], we created a database of 10,485,760 HCO simulated model instances (HCO-db, [32]) by systematically varying eight key parameters (a brute-force approach). The resulting parameter space includes 10,321,920 HCO instances which have at least one synaptic component present, and 163,840 isolated neuron instances which contain twin neurons without any synaptic interaction. By using our definitions above as criteria, we identified those simulated instances belonging to four groups [31]: functional HCOs encompassing 1,202,139 HCO instances and their subset of realistic HCOs having 99,066 instances, and of bursters encompassing 424 instances, of and their subset of realistic bursters encompassing 307 instances out of the entire database. By querying the HCO-db, we efficiently explored the instances from these four groups to determine which and how intrinsic membrane and synaptic parameters affect their electrical activity. In particular, we were interested in defining the parameter values that can lead to functional output from this circuit that conforms to that observed in the living system. For this, we applied the following methods to our groups of HCO model instances.

### Principal component analysis (PCA)

Principal component analysis (PCA) is a powerful tool used in many fields for identifying potentially hidden patterns within a large multidimensional data set. PCA, proposed by Pearson in 1901 [43], is a mathematical method based on an orthogonal linear transformation that allows for dimensionality reduction of a multidimensional data set without too much loss of information. This transformation converts the original data set into a set of linearly uncorrelated variables called principal components. Each principal component is a linear combination of the original variables. The number of principal components is less than or equal to the number of original variables. PCA allows for reducing the dimensionality of a data set by using only the first few principal components (considered the most important). The first principal component has the largest possible variance and each succeeding component in turn has the next highest variance possible and it is orthogonal to (i.e., uncorrelated with) the preceding components. Since all the principal components are orthogonal to each other, there is no redundant information.

We applied the principal component analysis (PCA) [43] to our four groups of interest. For this, we used the *princomp* function provided in the MATLAB Statistics Toolbox [44] to obtain the principal components (PC) for each group. This function returns the coordinates of the original data in the new coordinate system defined by the principal components. We can visualize each group of instances within the 3D space defined by the first three principal components obtained for the respective group (plots not shown, see S3: Figures 1–4). The *princomp* function also calculates the coefficients of the linear combinations of the original variables (our parameters) that generate the principal components. Each coefficient represents the importance of the respective parameter within the principal component (PC_i_  =  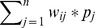, where n is the number of the original parameters p_j_, and w_i,j_ are the coefficients of these parameters for the principal component PC_i_). For each group of interest, we plotted the percent of variability explained by each principal component (see Results).

### 3D Orthogonal Regression Line

We used 3D Orthogonal Distance Regression (ODR) to assess the relationship between parameters identified in our PCA. Here we focus on the linear regression ([45]) and more precisely on the orthogonal linear regression method. The orthogonal linear regression method uses the Principal Components Analysis (PCA) method described above to fit a linear regression that minimizes the perpendicular distances from the data to the fitted model (least square fit is minimum). The method is also called Total Least Squares method or Principal Component Regression ([46]). Basically, given a set of points in a 3D space, the ODR method uses the coefficients of the first PCA corresponding to the 3D points to find a line (called 3D ODR line) in this 3D space that is least distance from the points. Once found, this line shows the tendency or direction of the points within the 3D space with respect to the three axes of the space.

### Two-sample Kolmogorov-Smirnov test

To test for the equality of our group distributions, we applied a two-sample Kolmogorov-Smirnov [47] nonparametric test (Figures 12A and 12B). To perform a two-sample Kolmogorov-Smirnov test on our distributions (see Results) we used the *kstest2* function in Matlab ([44]). The function was applied with the default Matlab values of ‘Alpha’  = 0.05 significance level and ‘Tail’ of ‘unequal’. We rejected the null hypothesis at the 5% significance level. The null hypothesis stated that the two samples are drawn from the same distribution.

## Supporting Information

Figure S1 **Plots of instances of a group in the 3D space defined by the first three main principal components obtained by applying PCA to the respective group.**
(DOC)

Figure S2 **Pairwise parameter variations for the realistic instances (HCOs and bursters).**
(DOC)

Figure S3 **5D clickable plots of HCOs and realistic HCOs.**
(DOC)

Figure S4 **Principal components for HCO and bursters groups.**
(DOC)

Table S1 **Coefficients of the linear combinations of the parameters that generate the principal components for each of the following groups of instances: HCOs, realistic HCOs, bursters isolated neurons, and realistic bursters.**
(DOC)

Text S1 **Supplementary Methods.** 3D Orthogonal regression line (ODR) and its cartesian form.
(DOCX)
