# Supplementary material for: Identifying Crucial Parameter Correlations Maintaining Bursting Activity
Source: PLoS Comput Biol. 2014 Jun 19;10(6):e1003678. doi: 10.1371/journal.pcbi.1003678 (PMC4063674; doi:10.1371/journal.pcbi.1003678)
Supplement: Figure S1 — Plots of instances of a group in the 3D space defined by the first three main principal components obtained by applying PCA to the respective group. (DOC) [file pcbi.1003678.s001.doc]

**Figure S1**

**Plots of instances of a group in the 3D space defined by the first three main principal components obtained by applying PCA to the respective group**

Figure S1A: **HCOs**

Figure S1B: **Realistic HCOs**


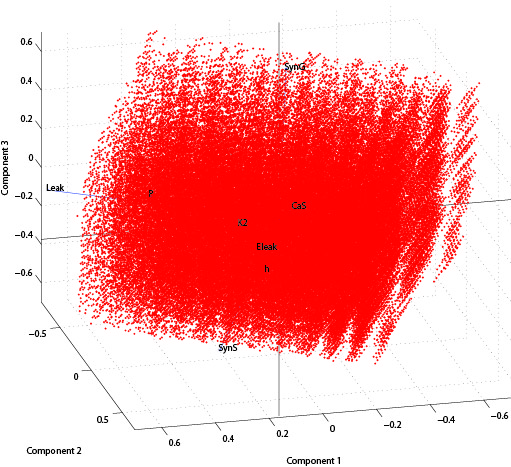


Figure S1C: **Bursters**

Figure S1D: **Realistic bursters**
