# Supplementary material for: Identifying Crucial Parameter Correlations Maintaining Bursting Activity
Source: PLoS Comput Biol. 2014 Jun 19;10(6):e1003678. doi: 10.1371/journal.pcbi.1003678 (PMC4063674; doi:10.1371/journal.pcbi.1003678)
Supplement: Figure S2 — Pairwise parameter variations for the realistic instances (HCOs and bursters). (DOC) [file pcbi.1003678.s002.doc]

**Figure S2**

**Pairwise parameter variations for the realistic instances (HCOs and bursters).**

Figure S2A: **Pairwise parameter variations for the realistic HCO instances.** The first parameter is shown on x axis. The second parameter is color coded. The number of instances within the group that have a particular parameter value is shown on the y axis. (**A**) There were instances within the group that have ḡ_h_ = 0%. For each E_Leak_ value, there is an amount of ḡ_h_ for which there is a clear peak in the number of instances. (**B**) The plot for ḡ_K2_ and ḡ_P_ conductances shows that these two conductances interact in producing realistic HCO instances. ḡ_P_ determined the peak number of the instances possible for this group and ḡ_K2_ determined what percentage of this peak was realized. However, there was an absolute peak in the number of instances for ḡ_K2_ = 125% and ḡ_P_ = 75% (7,773 instances), then the peaks (all occurred at same ḡ_P_ = 75%) decreased with increasing ḡ_K2_. For the HCOs, the peaks increased with increases in both ḡ_P_ and ḡ_K2_ (data not shown). (**C**) Another pair of conductances, ḡ_CaS_ and ḡ_Leak_, affected the number of realistic HCO instances. For any ḡ_Leak_ value, there was a peak number of instances at ḡ_CaS_ = 75%. The peak values in the number of realistic HCO instances decreased with increasing ḡ_Leak_. The largest number of instances occurred when ḡ_Leak_ =25%. (**D**) The plot for the pair of synaptic components reveals that the largest number of instances were obtained when ḡ_SynG_= 0%, i.e., there was no graded synaptic component or a very small amount of it (25%) and a large spike-mediated conductance (ḡ_SynS_ = 175%). For the HCOs (data not shown), increasing the strength of the graded synaptic component (ḡ_SynG_) increased the number of HCO instances. The largest number of HCOs was obtained for ḡ_SynG_ = 175% and ḡ_SynS_ = 75%.

Figure S2B: **Pairwise parameter variations for the realistic burster instances.** The first parameter is shown on x axis. The second parameter is color coded. The number of instances within the group that have a particular parameter value is shown on the y axis. (**A**) A non-zero ḡ_h_ was required to produce realistic burster instances. A peak in number of instances (28) was obtained for ḡ_h_= 150% and more negative values of E_leak_ (-70, -65 mV). (**B**) The peak number (16) of instances was produced when ḡ_K2_ = 25 and ḡ_P_ = 0%. (**C**) If ḡ_Leak_ = 0%, then at least 50% of ḡ_CaS_ was necessary to produce realistic burster instances. The peak in number of instances (14) was obtained when both ḡ_Leak_ = 175% and ḡ_CaS_ = 175%. Because each of the curves shown in these 3 plots has a different shape it is unlikely that there is simple pairwise relationship between the parameters for this group.
