# Supplementary material for: Identifying Crucial Parameter Correlations Maintaining Bursting Activity
Source: PLoS Comput Biol. 2014 Jun 19;10(6):e1003678. doi: 10.1371/journal.pcbi.1003678 (PMC4063674; doi:10.1371/journal.pcbi.1003678)
Supplement: Figure S3 — 5D clickable plots of HCOs and realistic HCOs. (DOC) [file pcbi.1003678.s003.doc]

**Figure S3**

**5D clickable plots of HCOs and realistic HCOs (points are not clicked)**

1. **HCOs group**

Figure S3A: **The axes of the 4D space are given by the ḡ_CaS_, E_Leak_, ḡ_K2_, and ḡ_P_ parameters.**

Figure S3B: **The axes of the 4D space are given by the ḡ_CaS_, ḡ_Leak_, ḡ_P_, and ḡ_SynS_ parameters**.

Figure S3C: **The axes of the 4D space are given by the ḡ_SynG_, ḡ_CaS_, ḡ_P_, and ḡ_SynS_ parameters.**

Figure S3D: **The axes of the 4D space are given by the ḡ_h_, ḡ_Leak_, ḡ_SynG_, and ḡ_K2_ parameters.**

Figure S3E: **The axes of the 4D space are given by the ḡ_K2_, ḡ_P_, ḡ_h_ and ḡ_Leak_ parameters.**

Figure S3F: **The axes of the 4D space are given by the ḡ_SynS_, ḡ_SynG_, E_Leak_ and ḡ_CaS_ parameters.**

1. **Realistic HCOs group**

Figure S3G: **The axes of the 4D space are given by the ḡ_CaS_, ḡ_K2_, E_Leak_, and ḡ_P_ parameters.**

Figure S3H: **The axes of the 4D space are given by the ḡ_SynG_, ḡ_SynS_, ḡ_h_, and ḡ_Leak_ parameters.**

Figure S3I: **The axes of the 4D space are given by the ḡ_Leak_, ḡ_P_, ḡ_SynS_, and ḡ_CaS_ parameters.**

Figure S3J: **The axes of the 4D space are given by the ḡ_h_, ḡ_Leak_, ḡ_SynG_, and ḡ_K2_ parameters.**

Figure S3K: **The axes of the 4D space are given by the ḡ_K2_, ḡ_P_, ḡ_h_ and E_Leak_ parameters.**

Figure S3L: **The axes of the 4D space are given by the ḡ_SynS_, ḡ_h_, ḡ_CaS_ and ḡ_SynG_ parameters.**
