# Supplementary material for: Identifying Crucial Parameter Correlations Maintaining Bursting Activity
Source: PLoS Comput Biol. 2014 Jun 19;10(6):e1003678. doi: 10.1371/journal.pcbi.1003678 (PMC4063674; doi:10.1371/journal.pcbi.1003678)
Supplement: Figure S4 — Principal components for HCO and bursters groups. (DOC) [file pcbi.1003678.s004.doc]

**Figure S4**

**Principal components (PCs) and variance explained by them.**

Figure S4A: **HCOs**

Figure S4B: **Bursters**
