# Supplementary material for: Identifying Crucial Parameter Correlations Maintaining Bursting Activity
Source: PLoS Comput Biol. 2014 Jun 19;10(6):e1003678. doi: 10.1371/journal.pcbi.1003678 (PMC4063674; doi:10.1371/journal.pcbi.1003678)
Supplement: Table S1 — Coefficients of the linear combinations of the parameters that generate the principal components for each of the following groups of instances: HCOs, realistic HCOs, bursters isolated neurons, and realistic bursters. (DOC) [file pcbi.1003678.s005.doc]

**Table S1**

**Coefficients of the linear combinations of the parameters that generate the principal components**

Table S1A. **HCO group**

| Param/PC | PC_1_ | PC_2_ | PC_3_ | PC_4_ | PC_5_ | PC_6_ | PC_7_ |
| --- | --- | --- | --- | --- | --- | --- | --- |
| E_Leak_ | -0.0292 | -0.0371 | 0.0343 | 0.1133 | 0.3518 | 0.3187 | 0.8311 |
| ḡ_P_ | 0.5857 | 0.1088 | 0.0610 | -0.0930 | -0.3356 | -0.1912 | 0.0336 |
| ḡ_K2_ | 0.2576 | 0.0789 | 0.2337 | 0.0677 | -0.6608 | 0.2710 | 0.3264 |
| ḡ_Leak_ | 0.7440 | 0.0779 | -0.1713 | -0.0975 | 0.5031 | 0.0700 | -0.0736 |
| ḡ_SynS_ | 0.1745 | -0.5461 | -0.0028 | 0.7440 | -0.0201 | 0.2302 | -0.2316 |
| ḡ_CaS_ | -0.0453 | 0.1767 | 0.0505 | -0.2631 | -0.0006 | 0.8487 | -0.3544 |
| ḡ_h_ | -0.0302 | -0.0863 | -0.9445 | -0.0626 | -0.2685 | 0.1000 | 0.1161 |
| ḡ_SynG_ | -0.0531 | 0.7985 | -0.1282 | 0.5811 | 0.0356 | -0.0123 | -0.0585 |

Table S1B. **Realistic HCO group**

| Param/PC | PC_1_ | PC_2_ | PC_3_ | PC_4_ | PC_5_ | PC_6_ |
| --- | --- | --- | --- | --- | --- | --- |
| E_Leak_ | -0.1086 | -0.1312 | 0.0828 | 0.0859 | 0.5346 | 0.2863 |
| ḡ_P_ | -0.4384 | 0.1748 | -0.0933 | 0.0566 | -0.4130 | -0.1695 |
| ḡ_K2_ | -0.1537 | -0.0271 | 0.0009 | 0.2896 | -0.6408 | 0.3648 |
| ḡ_Leak_ | -0.7684 | 0.4007 | -0.0655 | -0.1035 | 0.2942 | 0.0285 |
| ḡ_SynS_ | -0.3932 | -0.7438 | 0.3044 | 0.2598 | 0.0450 | 0.0937 |
| ḡ_CaS_ | 0.1168 | 0.2752 | 0.0765 | 0.0184 | 0.0271 | 0.8318 |
| ḡ_h_ | -0.1066 | -0.2461 | 0.1479 | -0.9081 | -0.2025 | 0.1644 |
| ḡ_SynG_ | -0.0479 | -0.3184 | -0.9272 | -0.0484 | 0.0526 |  |

Table S1C. **Bursters group**

| Param/PC | PC_1_ | PC_2_ | PC_3_ | PC_4_ |
| --- | --- | --- | --- | --- |
| E_Leak_ | 0.1462 | -0.0610 | 0.0180 | 0.5647 |
| ḡ_P_ | -0.6037 | 0.1076 | -0.1696 | 0.0022 |
| ḡ_K2_ | -0.4573 | -0.3912 | -0.1821 | -0.5133 |
| ḡ_Leak_ | -0.6091 | 0.1212 | 0.0252 | 0.5747 |
| ḡ_CaS_ | -0.0415 | -0.8272 | 0.4451 | 0.1834 |
| ḡ_h_ | -0.1797 | 0.3644 | 0.8596 | -0.2319 |

Table S1D. **Realistic bursters group**

| Param/PC | PC_1_ | PC_2_ | PC_3_ | PC_4_ |
| --- | --- | --- | --- | --- |
| E_Leak_ | 0.1340 | -0.0886 | 0.0474 | 0.4775 |
| ḡ_P_ | -0.6065 | 0.0947 | -0.2034 | 0.0050 |
| ḡ_K2_ | -0.4439 | -0.4119 | -0.1733 | -0.5664 |
| ḡ_Leak_ | -0.6086 | 0.0902 | 0.0199 | 0.5944 |
| ḡ_CaS_ | -0.0360 | -0.7984 | 0.4880 | 0.1732 |
| ḡ_h_ | -0.2133 | 0.4098 | 0.8294 | -0.2606 |
