# Supplementary material for: Identifying Crucial Parameter Correlations Maintaining Bursting Activity
Source: PLoS Comput Biol. 2014 Jun 19;10(6):e1003678. doi: 10.1371/journal.pcbi.1003678 (PMC4063674; doi:10.1371/journal.pcbi.1003678)
Supplement: Text S1 — Supplementary Methods. 3D Orthogonal regression line (ODR) and its cartesian form. (DOCX) [file pcbi.1003678.s006.docx]

**Text S1**

**3D Orthogonal Regression Line (ODR)**

Given a set of points in a 3D space with (x,y,z) coordinates of the points, there is a 3D line called the 3D Orthogonal Distance Regression (ODR) line such that is least distant from the points. The ODR line has the following characteristics. It contains the centroid of points, which is the 3D point that has the mean of the points on the three axes. Then, the direction vector that defines the line is given by the coefficients for the first principal component (PC). The second and third PCs are orthogonal to the first, and their coefficients define directions that are perpendicular to the ODR line. The simplest equation to describe the line is $Y=C+t*dirVect,$

where t parameterizes the position along the line, and C is the centroid with $C=\left( mean\left( X_{i} \right),mean\left( Y_{i} \right),mean\left( Z_{i} \right) \right)=\left( \overline{x},\overline{y},\overline{z} \right).$ Then, $dirVect=coeff\left( 1 \right),$ where coeff(1) is the first column of the coefficients returned by the PCA when applied to the set of 3D points (see [34] for method explanation, and [35] for an example).

It is possible to transform the parameterized form of the 3D line into its Cartesian (initial) form

$Ax+By+Cz+D=0,$ with A, B, C, D coefficients, by calculating these coefficients. Let $P_{1}=(x_{1},y_{1},z_{1})$ and $P_{2}=(x_{2},y_{2},z_{2})$ be two points on the line that are not the centroids. In particular, we can chose these points (as we did in our script) as the extreme points of the line, i.e., $P_{1}=(x_{min},y_{min},z_{min})$ and $P_{2}=(x_{max},y_{max},z_{max})$. Then, the coefficients are:

$$A=y_{min}-y_{max}+z_{min}-z_{max},$$

$$B=x_{min}-x_{max},$$

$$C=x_{min}-x_{max},$$

$$D=\left( x_{min}-x_{max} \right)*\left( \overline{y}+\overline{z} \right)-\overline{x}*\left( y_{min}-y_{max}+z_{min}-z_{max} \right).$$
